# The effectiveness of digital interventions for increasing physical activity in individuals of low socioeconomic status: a systematic review and meta-analysis

**DOI:** 10.1186/s12966-021-01218-4

**Published:** 2021-11-09

**Authors:** Max J. Western, Miranda E. G. Armstrong, Ishrat Islam, Kelly Morgan, Una F. Jones, Mark J. Kelson

**Affiliations:** 1 Centre for Motivation and Health Behaviour Change, Department for Health, University of Bath, Claverton Down, Bath, BA2 7AY UK; 2 Centre for Exercise, Nutrition and Health Science, School for Policy Studies, University of Bristol, 8 Priory Road, Bristol, BS8 1TZ UK; 3 PRIME Centre Wales, School of Medicine, Cardiff University, Cardiff, CF14 4YS UK; 4 Centre for Development, Evaluation, Complexity and Implementation in Public Health Improvement (DECIPHer), School of Social Sciences, Cardiff University, Cardiff, CF10 3BD UK; 5 School of Healthcare Sciences, College of Biomedical and Life Sciences, Cardiff University, Cardiff, CF14 4XN UK; 6 Department of Mathematics/Institute of Data Science and Artificial Intelligence, University of Exeter, Laver Building, Exeter, EX4 4QE UK

**Keywords:** Physical activity, Health inequalities, Behaviour change, RCT, Digital health, eHealth, Digital intervention, Socioeconomic status

## Abstract

**Background:**

Digital technologies such as wearables, websites and mobile applications are increasingly used in interventions targeting physical activity (PA). Increasing access to such technologies makes an attractive prospect for helping individuals of low socioeconomic status (SES) in becoming more active and healthier. However, little is known about their effectiveness in such populations. The aim of this systematic review was to explore whether digital interventions were effective in promoting PA in low SES populations, whether interventions are of equal benefit to higher SES individuals and whether the number or type of behaviour change techniques (BCTs) used in digital PA interventions was associated with intervention effects.

**Methods:**

A systematic search strategy was used to identify eligible studies from MEDLINE, Embase, PsycINFO, Web of Science, Scopus and The Cochrane Library, published between January 1990 and March 2020. Randomised controlled trials, using digital technology as the primary intervention tool, and a control group that did not receive any digital technology-based intervention were included, provided they had a measure of PA as an outcome. Lastly, studies that did not have any measure of SES were excluded from the review. Risk of Bias was assessed using the Cochrane Risk of Bias tool version 2.

**Results:**

Of the 14,589 records initially identified, 19 studies were included in the final meta-analysis. Using random-effects models, in low SES there was a standardised mean difference (SMD (95%CI)) in PA between intervention and control groups of 0.06 (− 0.08,0.20). In high SES the SMD was 0.34 (0.22,0.45). Heterogeneity was modest in both low (I^2^ = 0.18) and high (I^2^ = 0) SES groups. The studies used a range of digital technologies and BCTs in their interventions, but the main findings were consistent across all of the sub-group analyses (digital interventions with a PA only focus, country, chronic disease, and duration of intervention) and there was no association with the number or type of BCTs.

**Discussion:**

Digital interventions targeting PA do not show equivalent efficacy for people of low and high SES. For people of low SES, there is no evidence that digital PA interventions are effective, irrespective of the behaviour change techniques used. In contrast, the same interventions in high SES participants do indicate effectiveness. To reduce inequalities and improve effectiveness, future development of digital interventions aimed at improving PA must make more effort to meet the needs of low SES people within the target population.

**Supplementary Information:**

The online version contains supplementary material available at 10.1186/s12966-021-01218-4.

## Background

Physical activity (PA) incurs a multitude of health benefits and is consequently a cost-effective public health strategy for reducing the burden of non-communicable diseases [1]. The World Health Organisation reports that increasing physical activity levels worldwide could prevent 5 million premature deaths per year [2]. Exceeding the minimum recommended levels of PA can reduce the risk of colon and breast cancers, heart disease, stroke and diabetes by 20–30% [3, 4]. Recent estimates suggest that physical inactivity costs INT$54 billion to health care systems across the world, of which around 80% is incurred by high-income countries [5]. Despite a surge in promotion efforts over the past two decades the prevalence of physical inactivity increased between 2001 and 2016 from 32 to 37% in high-income countries and remained twice as high as that in low-income countries [6].

Socioeconomic status (SES) is a term used to describe an individual’s affluence or social standing, referencing factors such as wealth, educational level and occupation [7]. Many observers have found SES to associate with disparities in health and health behaviours, both within- and between-countries [8]. Globally, the life expectancy of a country’s population can range from 52 years in the poorest countries to 84 years in the richest [9]. The main drivers of this inequality are thought to be discrepancies in education, income, and access to medicine, care and health information [10]. The same factors also predict life expectancy within a given country, with some of the richest countries demonstrating considerable discrepancies in terms of morbidity and mortality rates between high and low SES groups [11]. This pattern is also true for PA behaviour; in a recent UK survey, around 50% of adults in the most deprived quintile met the PA recommendations compared to 68% in the least deprived quintile [12]. Indeed, around the world, SES is thought to have a strong positive relationship with leisure-time PA [13–16], which is considered the PA domain best associated with overall health benefits [17, 18].

The rapid growth in number and sophistication of digital technologies such as websites, mobile or wearable devices, smartphone applications and telehealth or telemedicine have been presented as a cost-effective platform for promoting PA behaviour change and health improvement [19–21]. Access to such technologies are increasing around the globe, with internet penetration as high as 95% in the most developed nations and 60% worldwide [22]. Indeed, in the USA and UK over 90% of all adults own a smart phone, rising to around 95 and 99% in 35–55- and 16–34-year old’s respectively [23] implying that in these countries a large majority of the population across SES currently use such technologies. Digital technologies enable researchers and clinicians to develop remote interventions that are grounded in behavioural theory [24], can include a number of potentially useful behaviour change techniques [25] and can be tailored to meet the particular needs of a given individual or population [26]. Accordingly, digital technologies have been championed as a vehicle that reduces health inequalities by taking bespoke, informative and empowering programmes to otherwise hard to reach, low SES, populations [27].

Others, however, have argued that the use of digital technologies for health [behaviour] promotion can in fact create a ‘digital divide’ and that wearables and smartphone or web applications are predominantly designed for more affluent (and higher SES) people with higher levels of education and income [28, 29]. In particular, one’s eHealth literacy (the term used to describe an individual’s ability to seek out, comprehend, critique and act upon health-related knowledge and guidance delivered through digital means) is an important factor that can determine whether or not simply having the access to digital technologies promoting health behaviour change is actually useful to an individual [30–32]. A number of studies have demonstrated that low SES individuals tend to also have lower eHealth literacy and consequently do not incur the same benefits as their higher SES counterparts when engaging with digital health technologies [33–35].

Little is currently known about whether interventions deploying digital technologies to increase PA are equally effective in high and low SES populations. Systematic reviews indicate that the general effectiveness of eHealth interventions on PA behaviour change is modest yet promising [36–40]. However, neither the individual randomised controlled trials nor the pooled analysis included in these systematic-reviews have analysed their data in a way that separates the effects observed in higher and lower SES groups. This is important, as even digital behaviour change interventions that demonstrate a net overall effect on PA behaviour when comparing the intervention and control groups, may risk silently exacerbating health inequalities if its programme is effective for high SES populations but makes no difference to low SES populations. Moreover, analysing study populations as a whole does not inform us if the behaviour change techniques used in digital interventions are ubiquitously useful across SES groups. Behaviour change techniques (BCTs) refer to the active ingredients of a given intervention that aim to evoke a change in behaviour, which have been classified according to their nature such as *goal setting*, *feedback and monitoring*, and *shaping knowledge* [41]. Systematic reviews of PA interventions in other contexts have shown that the number of BCTs, which may be a marker of intervention complexity, does not necessarily dictate how effective an intervention may be [42, 43] but have not analysed their data according to SES. Understanding whether interventions have varying effectiveness for individuals of low SES compared to higher SES, and if any number or type of particular BCTs is particularly useful for low SES populations would help researchers and policymakers appropriately tailor their efforts towards reducing inequalities in PA promotion.

Accordingly, the aim of this systematic review and meta-analysis is to understand whether digital behaviour change interventions targeting increased PA are beneficial for low SES populations. Specifically, we set out to investigate the following research questions:

1. Are digital behaviour change interventions effective at promoting a change in PA behaviour when comparing the intervention with control groups amongst low SES participants?
2. Do digital behaviour change interventions promoting PA have equivalent effectiveness when we compare the effects of the intervention versus control group in high SES participants to the effects found in low SES participants [identified in research question 1]?
3. Is the number or type of behaviour change techniques (BCTs) included in digital behaviour change interventions promoting PA associated with the study outcome in low and high SES groups?

## Methods

The protocol for this systematic review is registered with the international prospective register for systematic reviews (PROSPERO, registration ID: CRD42018079540). The design and implementation of this review conform to the Preferred Reporting Items for Systematic Reviews and Meta-analyses (PRISMA) guidelines (see Supplementary File 3 for Checklist).

### Eligibility criteria

The population, intervention, comparison, outcome (PICO) framework was used to develop the inclusion and exclusion criteria for study selection in this review. The *population* of interest was any human study with participants aged between 0 and 100 years. Studies were excluded if the targeted populations are with rare diseases, defined as having a prevalence of 1 in 2000 persons [44]. Studies were also excluded if there was no index of SES status (e.g. SES index, income, education, employment) used to characterise the participants. *Interventions* were included if they adopted an RCT design (including cluster RCT) as we view this as the best way to identify causally valid and homogenous studies, and used a digital technology, which we operationalise as *any web-based interface or wearable device that communicates information to the user, any mobile-based program, or offline-computer program*, as the primary intervention tool. Studies were excluded if they contained a pharmacological component alongside the digital technology within an intervention, or if thegital technology was secondary to a therapeutic, face-to-face or counselling based intervention. As the focus of the review was on the effectiveness of digital technology, studies were only included if the *comparator* group did not receive any digital technology-based intervention. Lastly, studies that did not have any measure of PA (such as time in moderate to vigorous intensity, steps or sedentary time) as an *outcome* were excluded from the review.

### Search strategy

A combination of terms relating to or describing the intervention was used to run the search. The search period was from January 1990 until March 2020 as it was assumed that any study which pre-dated this point in time would not be generalisable to our current understanding of digital interventions. All authors contributed to the development of the search strategy, and the full list of search terms has been provided in Supplementary File 1. The search was conducted by an expert subject librarian using MEDLINE, Embase, PsycINFO, Web of Science (Science and Social Science Citation Index), Scopus and The Cochrane Library (Cochrane Database of Systematic Reviews, Cochrane Central Register of Controlled Trials (CENTRAL), Cochrane Methodology Register). The search terms within MEDLINE and Embase included a filter for controlled trials of interventions. Reference lists from relevant systematic reviews and meta-analyses were searched to identify any additional studies. Where relevant protocol papers were identified during the search, an attempt was made to find the accompanying trial papers. Only papers published or available in English language were considered.

### Data extraction

Data were reviewed and extracted in pairs formed by five members of the team (MW, MA, II, KM, UJ). Initially, titles were checked for relevance and the abstracts of the relevant titles were screened. Partial data extraction was conducted from the full-texts of the relevant abstracts to assess the inclusion and exclusion criteria. Detailed data extraction was then conducted from the full texts of the included studies. At each stage, two authors independently reviewed the titles, abstracts and full-texts to include or exclude them for the next stage. Any disagreements were resolved via group discussion. With regards to research question 3, behaviour change techniques (BCTs), which were coded according to the comprehensive definitions found in Michie et al.’s BCT Taxonomy v1 [41], were extracted by two independent reviewers (MW and II) for each analysed study, with any disagreements resolved via group discussion with the full authorship team. The data extraction form can be found in Supplementary File 1.

### Data analysis

Quantitative data for meta-analysis were identified either through extraction from published manuscripts, or through requesting additional summary statistics from authors, or by requesting individual participant data from authors and constructing our own summary statistics. The study team created a hierarchy of preferred metrics for both SES (1. Specific SES measure or index of deprivation; 2. Income; 3. Education; 4. Employment) and PA (1. minutes of Moderate-to-Vigorous-Intensity PA, 2. Total PA minutes, 3. Steps, 4. Sedentary time). Where studies reported multiple SES measures (e.g. education and income) or PA outcomes (e.g. steps and MVPA) in their manuscripts, a request to authors was made in line with the highest-ranking metric of interest in this hierarchy. Definitions for what constituted low and medium/high SES was decided on a study-by-study basis based on the measures reported and what was appropriate for the context and study (for example country of origin and whether continuous or categorical scales were used to collect the data). For deprivation indices decile or quintile cut points were most often reported. For education most authors used a split between pre-university education and university educated or higher. Income splits differed by currency and year of publication, but a median household cut point was most frequently used. Employment was used more rarely (one study) and was split by manual or intermediate versus higher managerial. Table 1 indicates the specific definitions for each study. Authors of potentially relevant papers (*n* = 49) were contacted twice and given a minimum of 2 weeks each time to respond to requests for additional information. Thirty authors were either unable to provide data or did not respond to the request.

**Table 1:** Summary of included studies

Author, date, setting, location, study design	Population/health condition, age, gender	SES High/low and % per group	Intervention: type of tech, duration, frequency, follow-up, additional support/incentives	Number of behavioural change techniques used	Outcome measure and measuring tool.
Aittasalo 2012 [45] Office-based occupational health units, Southern Finland, RCT.	Insufficiently physically active healthy participants. I: *n* = 123, 44.1 yrs. 87% female C: *n* = 118, 45.3 yrs. 78% female	Education: High: University, polytechnic I = 94%, C = 91%. Low: basic I = 6%, C = 9%.	I: One-hour preliminary meeting including benefits of PA and walking, self-monitoring of PA with a pedometer (Omron, Walking Style II) and logbooks, monthly email message. C: No intervention. Duration: 12 months.	7	Walking minutes/week (to work, for transport, for leisure, stairs), sedentary time. (SR)
Alley 2016 [46] Australian metropolitan and regional cities, RCT.	Adults not meeting physical activity recommendations. I1: *n* = 53; 55.3 yrs. 67% female I2: *n* = 56; 52.2 yrs. 75% female C: *n* = 45; 55.2 yrs. 84% female	Income/year: High >AUD$52 K I1 = 68%, I2 = 62%, C = 69%. Low <AUD$52 K I1 = 32%, I2 = 38%, C = 31%	I1: ‘My Activity Coach’ web-based intervention delivered 1 module of computer-tailored advice every 2 weeks. Biweekly web-based video coaching. (Alley 2.0 in analysis) I2: ‘My Activity Coach’ web-based intervention delivered 1 module of computer-tailored advice every 2 weeks. Biweekly email reminder. (Alley 1.0 in analysis) C: No intervention, waiting list. Duration: 8 weeks, follow up 6 months. The incentive to those who complete all surveys: entry into the draw for a pedometer, Fitbit, heart rate monitor.	10	PA minutes/week via Active Australia Survey. (SR)
Ashton 2017 [47] Hunter region, New South Wales, Australia, RCT.	Men aged 18–25 not meeting physical activity recommendations.I: *n* = 26, 22.4 yrs. C: *n* = 24, 21.9 yrs	Income/week: High > $1 K I = 15%, C = 4% Low < $1 K I = 85%, C = 96%	I: A website with resource library, Jawbone PA tracker with an associated mobile phone app, One-hour weekly face to face group sessions, personalised food and nutrient report, private Facebook discussion group, Gymstick resistance band, TEMPlate guide for meal portions. C: No intervention, waiting list. Duration: 3 months.	9	Steps/day measured by Yamax digiwalker SW200 pedometers. Minutes/week MVPA using Godin leisure-time exercise questionnaire. (SR)
Creel 2016 [48] Midwestern US city, RCT.	People undergoing bariatric surgery. I1: *n* = 52, 41.8 yrs. 84.6% female I2: *n* = 48, 43.6 yrs. 83.3%female C: *n* = 50, 44.2 yrs. 84% female	Years of Education: High > 14 yrs.; I1 = 37%, I2 = 36%, C = 36% Low ≤14 yrs. I1 = 63%, I2 = 64%, C = 64%	I1: Omron HJ-113 pedometer, data recorded by participants daily, written advice on how to increase PA, Educational booklet. I2: Omron HJ-113 pedometer, data recorded by participants daily, education manual, exercise counselling at clinic visits, Educational booklet. C: Educational booklet. Interventions were delivered before and for 6 months after bariatric surgery Duration: 6 months Incentive: Study completers received $50 gift card.	3	Steps/day, MVPA bouts, sedentary time, and light physical activity measured by GT3X accelerometer.
Duncan 2014 [49] Queensland, Australia, RCT	Males aged 35–54 years I: *n* = 205, 44.2 yrs. C: *n* = 96, 73.8 yrs	Education: High: Higher/further education I = 78.1%, C = 79.1%; Low: Secondary school or less I = 22%, C = 20.8%	I: IT-based ManUp challenges on PA and healthy eating C: Print-based ManUp challenges on PA and healthy eating Duration: 9 months.	11	PA minutes/week via Active Australia Questionnaire. (SR)
Fjeldsoe 2016 [50] New South Wales, Australia, RCT.	Healthy adult participants. I: *n* = 114, 55.5 yrs. 64.9% Female C: *n* = 114, 51.2 yrs. 68.4% female	Deprivation: High: SEIFA > lowest 2 quintiles I = 75%, C = 68% Low: SEIFA = lowest 2 quintiles I = 25%, C = 32%	I: Get Healthy, Stay healthy initiative - tailored text messages ranging from 4/fortnight to 1/fortnight. C: No intervention, written feedback from each assessment point. Duration: 6 months.	12	MVPA via Actigraph GT1M. Walking > 30 min (frequency), MVPA> 30 min (frequency) from 3Q-PA. (SR)
Golsteijn 2018 [51] Hospital oncology departments throughout The Netherlands, RCT.	Patients and survivors of prostate and colorectal cancer. I: *n* = 249, 66.6 yrs. 14.9% Female C: *n* = 229, 66.4 yrs. 10.9% Female	Education: High I = 56%, C = 50%; Low I = 44%, C = 50.0%	I: OncoActive intervention. Tailored PA advice at baseline, after 2 months, after 3 months delivered online and in paper; provision of pedometer and access to interactive online PA resources. C: Usual care. Duration: 3 months, follow-up at 6 months	9	MVPA minutes/week via Actigraph. MVPA days > 30 min of PA via Short Questionnaire to Assess Health Enhancing Physical Activity (SQUASH) (SR)
Greaney 2017 [52] Rural North Carolina, USA, RCT.	Overweight black females. I: *n* = 60, 36.6 yrs. C: *n* = 61, 35.6 yrs	Federal Poverty Level (FPL): High: Above FPL I = 38.9%, C = 35.6%; Low: At or below FPL I = 61.1, C = 64.4	I: Shape intervention - tailored behaviour change goals, printed skills training materials, weekly interactive voice responses telephone calls, monthly telephone coaching, a no-cost membership to YMCA facility. For the first 8 weeks, participants were assigned 3 goals, and then 4 goals for each 8-week interval. C: semi-annual newsletters on general wellbeing and not including PA, nutrition, and weight. Duration: 12 months.	10	MVPA minutes via Actical accelerometer.
Gutierrez-Martinez 2018 [53] Bogota, Columbia, Cluster RCT.	Schoolchildren I1: *n* = 57, 10.4 yrs. 57.9% female I2: *n* = 60, 10.4 yrs. 60.0% female C: *n* = 67, 10.6 yrs. 53.7% female	Socioeconomic group: High: Group 3 I1 = 35.1%, I2 = 3.3%, C = 6.0%; Low: Groups 1 and 2 I1 = 64.9%, I2 = 96.7%, C = 94.0%	I1: MARA+SMS MARA = Standardised recess activities 20 min, 3/week SMS = daily SMS sent to parents/children. Parents were asked to show children every SMS. I2: MARA. C: No intervention Duration: 10 weeks.	2	MVPA minutes/day and minutes/week, via Actigraph GT3X+.
Hawkins 2019 [54] 8 local authorities in Wales, UK, RCT.	Adults referred to a National Exercise Referral Scheme. I: *n* = 88, 55.1 yrs. 60% female C: *n* = 68, 58.5 yrs. 74% female	Income/year: High > £31 K I = 24%, C = 19% Low ≤£31 K I = 76%, C = 81%	I: Enhanced exercise referral programme inc. usual care plus an accelerometer-based activity monitor (MyWellnessKey) and MyWellnessCloud web portal. Usual care = 16-week structured exercise programme with consultations at start, 4 weeks, 16 weeks and 12 months. C: Usual care. Duration: 16 weeks, follow-up at 12 months	6	MVPA minutes, PA volume, sedentary behaviour via Actigraph GT3X. This data was collected from a subset of 53/99 of the study sample.
Houle 2011 [55] Regional hospital, Quebec, Canada, RCT.	Adults with Post-acute coronary syndrome I: *n* = 32, 58 yrs. 18.8% female C: *n* = 33, 59 yrs. 24.2% female	Family income: High > $30 K I = 75%, C = 75% Low ≤$30 K I = 25%, C = 25%	I: Provision of a pedometer (Yamax Digiwalker SW-200), diary and information regarding exercise after acute coronary syndrome, using the pedometer, and recommended exercise goals by a clinical nurse specialist. One phone call within 2 weeks, face to face consultations at 3, 6, 9, and 12 months C: Usual care. Duration: 12 months.	9	Steps/day by Yamax Digiwalker NL-2000.
Laing 2014 [56] Primary Care clinics, Los Angeles, USA, RCT.	Adults with BMI > 25. I: *n* = 107, 43.2 yrs. 76% female C: *n* = 105, 43.1 yrs. 70% female	Annual income: High: > $75 K I = 41%, C = 33% Low ≤$75 K I = 59%, C = 67%	I: Usual care plus Mobile Fitness project app. C: Usual care. Duration: 6 months. Incentive: $20 gift card for attending each follow-up visit.	6	PA days/week (SR)
Muller 2016 [57] Urban Malaysia, RCT.	Older adults. I: *n* = 22, 63.6 yrs. 73% female C: *n* = 21, 62.9 yrs. 76% female	Education: High: Post-secondary I = 73%, C = 81% Low: Secondary I = 27%, C = 19%	I: Booklet with information on benefits of exercise, safety, age-appropriate exercises. Daily text messages including instruction and praise. C: Booklet with information on benefits of exercise, safety, age-appropriate exercises. Duration: 12 weeks, follow up 24 weeks.	4	Exercise frequency (SR). Weekly MET-Minutes and daily sitting hours via IPAQ short form (SR).
Phelan 2017 [58] Women, Infants and Children clinics in California, USA, RCT.	Postpartum women with low-income. I: *n* = 174, 27.5 yrs. C: *n* = 196, 28.6 yrs	All sample are low SES.	I: Standard care plus internet-based weight loss programme including goal setting for weight and physical activity, online resources, automated feedback, web diary, weight, and physical activity tracker, instructional and inspirational videos, and message board. Four weekly text messages re new material, motivation, support, and feedback. Monthly face to face group sessions. C: Standard care plus newsletters every two months with information about weight control, exercise, nutrition, and wellness. Duration:12 months.	9	MVPA, LPA sedentary time minutes per day, via GT3X+ Actigraph.
Taylor 2016 [59] Four worksites, Texas, USA, Cluster RCT.	Desk-based working adults. I1: *n* = 59, 44 yrs. I2: *n* = 69, 43 yrs. C: *n* = 47, 42 yrs	Education: High: more than high school I1 = 97%, I2 = 91%, C = 85% Low: High school diploma or less I1 = 3%, I2 = 9%, C = 15%	I1: Computer prompts of 3-min breaks every hour over 5 h. Computer software Workrave v 1.10, Eyes relax v 0.87. Prompts encouraged getting up to walk hallways, stairs, outdoors. I2: One daily break of 13–15 min. Group peer-led sessions of stretching, strengthening, aerobic movements, and meditation. C: Usual breaks, no intervention. Typical patterns were 2 × 15-min breaks and 30–60 min for lunch. Duration: 6 months. Incentive: $25 for baseline and 6-month assessment, $50 for 12-month assessment.	2	Steps/week, steps/day via New Lifestyles DigiWalker SW200 pedometer. Physical activity MET minute/week and sedentary time MET minute/week via IPAQ long-form. (SR) Sedentary leisure time minute /day via Neighbourhood quality of life study. (SR)
Vallance 2007 [60] Northern Alberta, Canada, RCT.	Adult women breast cancer survivors. I1: *n* = 94, 58 yrs. I2: *n* = 93, 58 yrs. I3: *n* = 94, 57 yrs. C: *n* = 96, 57 yrs	Income: High: >$59 K I1 = 45.5%, I2 = 51.2%, I3 = 36.8%, C = 46.1% Low: ≤$59 K I1 = 54.5%, I2 = 48.8%, I3 = 63.2, C = 53.9%	I1: Pedometer (Digi-walker SW-200) and a 12-week step calendar. I2: Pedometer and Printed materials - exercise for Health: An exercise guide for Breast Cancer Survivors. I3: Printed materials - exercise for Health: An exercise guide for Breast Cancer Survivors. C: No materials, recommended to maintain PA guideline. Duration: 12 weeks.	2	Steps/day via Digi-walker pedometer. MVPA, VPA total minutes, walking frequency, and duration via the leisure score index of the Godin Leisure time Exercise Questionnaire. (SR)
Valle 2017 [61] Hospital clinics, local community North Carolina, USA, RCT.	Adult women African American breast cancer survivors with BMI 20–45. I1: *n* = 11, 52.2 yrs. I2: *n* = 13, 52.6 yrs. C: *n*= 11, 54.4 yrs	Education: High: College or higher I1 = 100%, I2 = 84.6%, C = 72.7%; Low: High school or less I1 = 0%, I2 = 15.4%, C = 27.3%	I1: Activity tracker (Withings Pulse) plus 1 face to face education session, Weighing scales with a companion app, 24 weekly emailed tailored feedback on weight and physical activity. I2: 1 face to face education session, Weighing scales and companion app, 24 weekly emailed tailored feedback on weight. C: 1 face to face education session, weighing scales with a companion app. Duration: 6 months. Incentive: $40 for completing each data collection point.	10	PA minutes/5 days, minutes/week via Paffenbarger Activity Questionnaire. (SR)
Van der Weegen, 2015 [62] Southern Netherlands, Cluster RCT.	People with Type 2 Diabetes and people with COPD who did not comply with Dutch norm for healthy exercise. I1: *n* = 65, 57.5 yrs. 52.3% female I2: *n* = 66, 56.9 yrs. 47.0% female C: *n* = 68, 59.2 yrs. 54.4% female	Education: High: I1 = 70.8%, I2 = 71.2%, C = 77.9% Low I1 = 29.2%, I2 = 28.8%, C = 22.1%	I1: Self-management support programme (SSP) and monitoring and feedback tool. SSP = four individual consultations with the practice nurse in week 1, after 2–3 months, and after 4–6 months. Monitoring and feedback tool = activity monitors mobile phone app and web app. I2: SSP only. C: Usual care. Duration: 6 months, follow-up 9 months.	5	PA minutes/day via Personal Activity Monitor AM 300.
Watson 2015 [63] Belfast, Northern Ireland, RCT	Overweight and obese adults. I: *n* = 32, 51.4 yrs. 50% female C: *n* = 33, 52.9 yrs. 61% female	Occupational class: (both groups) High: Class 1 = 51% Low: Class 2 & 3 = 49%	I: Imperative Health web-based programme (AXA PPP Healthcare Ltd) including monitoring, feedback. C: Usual care. Duration: 24 weeks, follow up 6 and 12 months.	12	MVPA minutes/day as measured by the Recent Physical Activity Questionnaire. (SR)

*RCT* Randomised Control Trial, *SR* Self-reported, *yrs.* years, *COPD* Obstructive Pulmonary Disease, *SEIFA* Socio-Economic Indexes for Areas

PA outcomes were extracted and combined using random-effects meta-analysis. This decision was made in advance of conducting meta-analysis and based on the expected heterogeneity in study designs, settings, interventions, populations, and time frames. Fixed effect meta-analysis is also reported as was pre-specified in our PROSPERO registration. Baseline and Follow-up scores in PA (or more rarely change scores) were extracted from papers or individual-level data. Measures of precision were extracted from standard errors, or standard deviations. For most of the studies included we were given access to the raw data and so we were able to calculate standard deviations directly. Where studies had multiple arms in their trial that were eligible for inclusion in the analysis, the control group was split equally between intervention arms to avoid double counting of participants.

The analysis for research questions 1 and 2 was performed in the R programming language and environment version 3.6.1 [64] and using the ‘meta’ package, [65]. I^2^ statistics were calculated for meta-analysis and forest plots produced. Where more than 10 studies were included in forest plots for our primary objectives a funnel plot was also produced to explore publication bias and Egger’s statistic for assessing publication bias was calculated [66]. Research question 3 was addressed using meta-regression using the ‘metareg’ package in R (version 4.15–1) using a single explanatory covariate (number of behaviour change techniques employed at a study level) in order to explore whether studies employing more behaviour change techniques were observed to have larger intervention effects. Bubble plots were used to summarise the findings.

Sub-group analysis was pre-specified for the following categories: digital interventions with a sole focus on PA versus other targets (e.g. weight loss), study setting (countries/continents), excluding studies at high risk of bias, age groups (Under 5, 5–18, Adult 19–64, > 64 years), healthy or general population/versus chronic disease populations, duration of the active intervention (less than 3 months, 3–6 months, greater than 6 months), duration of follow-up (less than 6 months, more than 6 months to 1 year, more than 1 year), and pregnancy. Cluster randomised trials had sample sizes adjusted to effective sample sizes accounting for average cluster sizes and Intracluster Correlation Coefficients (ICCs).

### Risk of bias assessment

Two authors (MW and KM) performed an independent assessment of the risk of bias on each of the included studies in line with the updated Cochrane Risk of Bias in randomised trials ‘RoB 2’ [67]. All studies were graded by both authors with disagreements being resolved in discussion with the wider research team. The scoring algorithms presented in the RoB 2 were used to determine the low, moderate, or high risk of bias against each of the core six criteria.

## Results

**Fig. 1:**
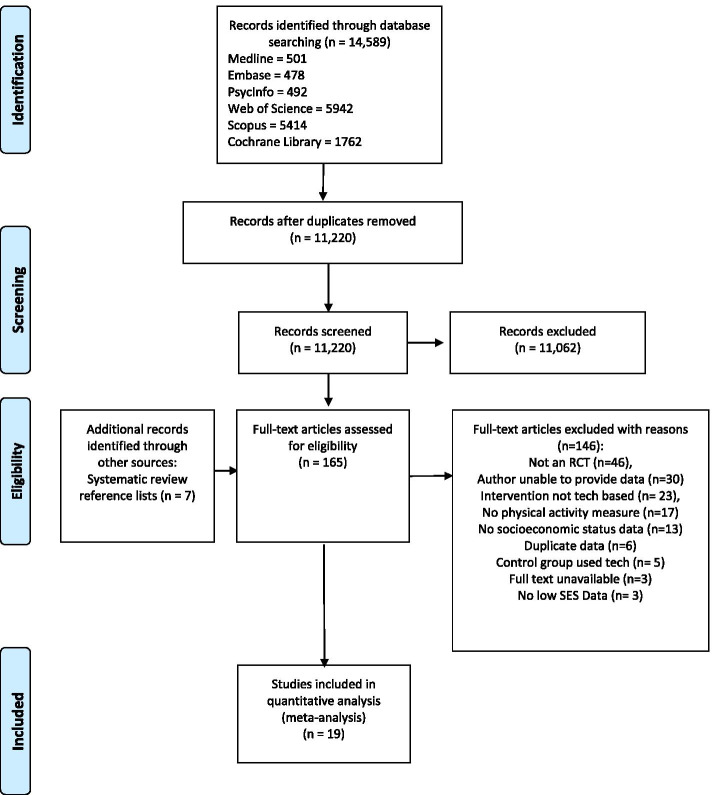
PRISMA flow diagram of study selection

A PRISMA diagram for the study selection process including reasons for exclusion is shown in Fig. 1.

### Study characteristics

**Fig. 2:**
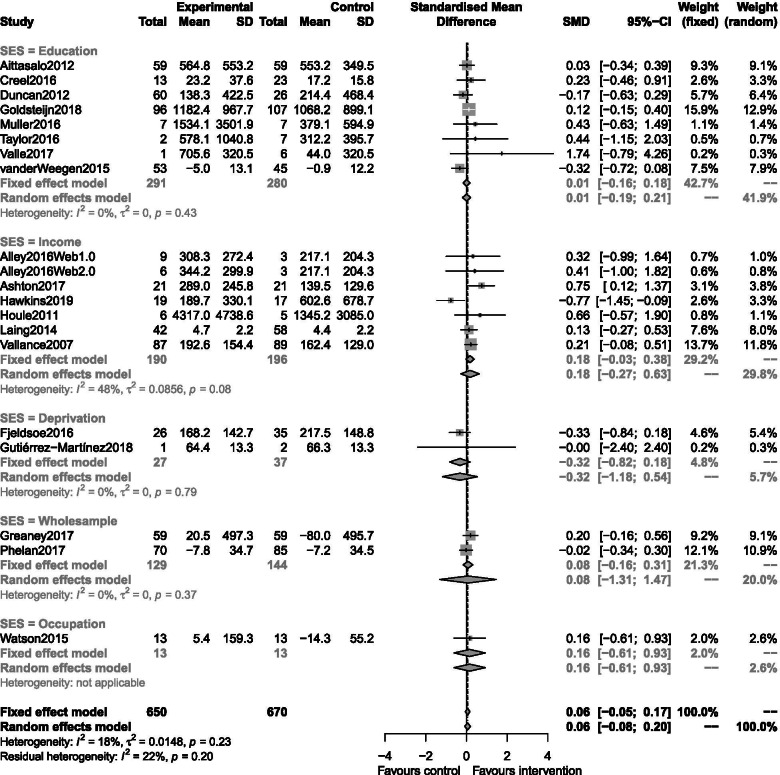
Forest plots depicting the pooled standardised mean difference across all reviewed studies included in the low SES meta-analysis by SES metric

**Fig. 3:**
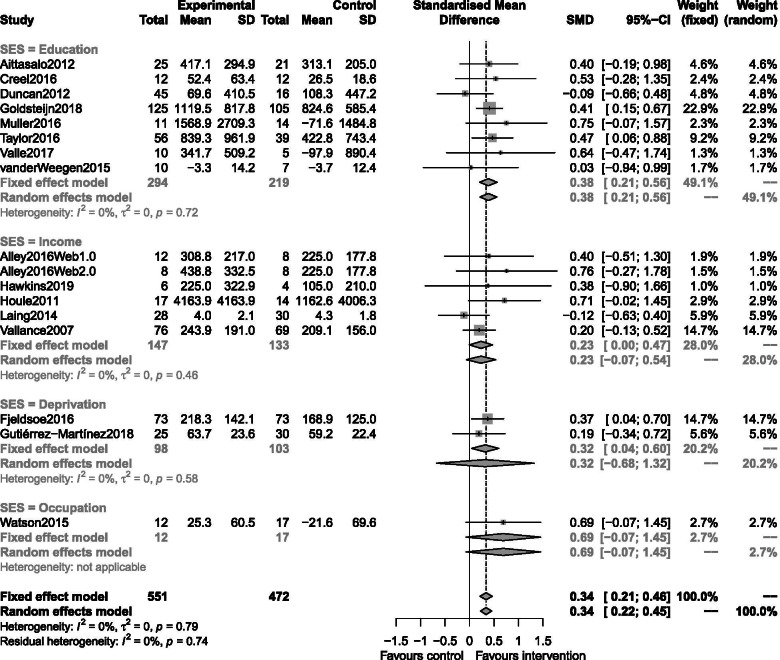
Forest plots depicting the pooled standardised mean difference across all reviewed studies included in the high SES meta-analysis by SES metric

The review included 19 studies comprising 16 RCT [45–52, 54–58, 60, 61, 63] and three cluster RCT [53, 59, 62], which are summarised in Table 1. The studies took place in North America (*n* = 8) [48, 52, 55, 56, 58–61], Europe (*n* = 5) [45, 51, 54, 62, 63], Australia (*n* = 4) [46, 47, 49, 50], Asia (*n* = 1) [57], and South America (*n* = 1) [53]. Eleven interventions were explicitly targeting PA behaviour [45, 46, 48, 51, 53–55, 57, 59, 60, 62], while eight were targeting weight loss, general health or multiple lifestyle behaviours (e.g. PA and diet) [47, 49, 50, 52, 56, 58, 61, 63]. The included studies used a number and a combination of digital technologies such as web-sites [46, 47, 49, 51, 54, 58, 59, 62, 63], activity trackers [45, 47, 48, 51, 52, 54, 55, 58, 60–63], text messaging or email feedback or prompts [45, 46, 50, 53, 57, 58, 61], and mobile applications [47, 49, 56, 61, 62] in their interventions. Interventions lasted between 8 weeks and 12 months, most common durations were 6 months (*n* = 5) and 12 months (*n* = 4) as described in Table 1. Outcome measures included a range and combination of PA assessment methods (Table 1). Using our hierarchy for prioritising outcome measure, our primary analysis involved ten studies using self-reported measure of MVPA [51, 60, 63], total physical activity [46, 49, 54, 56, 57, 61] or walking [45], and nine studies used device-based assessments of MVPA [47, 48, 50, 52, 53, 58, 62], leisure time PA [59], or steps [55] (Figs. 2 and 3).

Measurement of SES was recorded in different ways across studies, with some studies including a number of methods. In the meta-analysis, the best measure of SES was considered from the following pre-specified priority list: deprivation score (i.e. index of multiple deprivations, SES group or federal poverty line, *n* = 3 [50, 52, 53]), income (*n* = 6 [46, 47, 54–56, 60]), education (*n* = 9 [45, 48, 49, 51, 57–59, 61, 62]), employment (*n* = 1 [63]). Each SES was then dichotomised into date-adjusted high and low categories (Table 1). Socio-Economic Indexes for Areas was categorised as low if in the lower two quintiles. Federal poverty level was categorised as low if at or below the Federal Poverty Line. The median income for the specific country at the time of data collection was used as a cut-off between high and low income. Education was low if equivalent to 14 years or less (i.e. no higher education). SES group was categorised as low for the two lowest groupings used. While the included studies randomised 5419 participants between them, once we had accounted for attrition, data availability and a focus on relevant study arms we ended up with a sample of *n* = 1317 low SES and *n* = 1023 medium-high SES participants for analysis.

The three cluster randomised trials had their sample sizes scaled according to their average cluster sizes. Only one study [62] reported an ICC (of 0.005) and this was used for all three studies.

### RQ1 effectiveness of digital interventions on physical activity in low socioeconomic status groups

Twenty interventions from nineteen studies were included in this meta-analysis (one study, Alley et al., 2016 [46], appears twice as it was a three-arm trial). Interventions were grouped according to how they measured SES. Heterogeneity was low (I^2^ = 18%). There was little difference between the fixed and random-effects analysis. This analysis did not identify a statistically significant intervention effect in low SES groups (standardised random-effects estimate: 0.06, 95% CI [− 0.08,0.20]). A funnel plot did not indicate publication bias (*p* = 0.37).

### RQ2 equivalence of digital interventions on physical activity in low socioeconomic status groups

Seventeen interventions from sixteen studies were included in this meta-analysis conducted in high SES participants (again Alley 2016 appears twice, with a split control group), split by how SES was determined. Heterogeneity was low (I^2^ = 0%). Fixed and random-effects estimates did not differ substantially. This analysis identified a statistically significant effect of about a third of a standard deviation in favour of intervention for this group (standardised random-effects estimate: 0.34, 95% CI [0.22,0.45]). A funnel plot did not indicate publication bias (*p* = 0.45).

### Subgroup analysis for RQ1 and RQ2

We were able to conduct subgroup analysis for some of our pre-specified categorisations. These were: digital interventions with a PA only focus, country, chronic disease, and intervention length. We were not able to explore studies at high risk of bias (only two studies at high risk of bias provided data), age group (included age groups were too disparate), and pregnancy (only one study had a postpartum focus). Post-hoc subgroup analysis was also performed to explore whether there were any differences depending on objective versus self-report measures of PA, and whether the study used and active or passive control condition. None of our subgroup analyses indicated differential effects by subgroup (Supplementary File 2, Appendices A and C).

### RQ3 what behaviour change techniques are most effective in low SES groups?

**Fig. 4:**
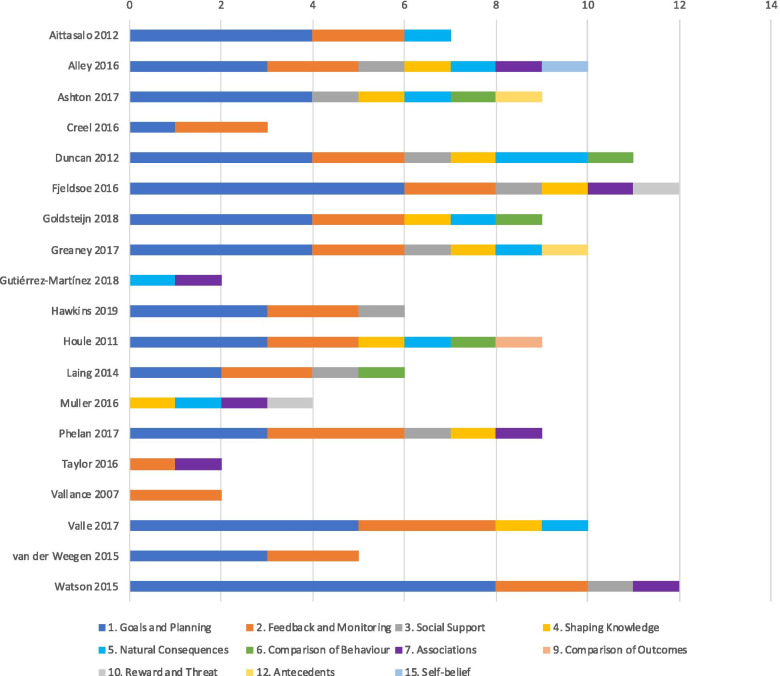
Behaviour Change Techniques used in included studies. The stacked bars represent the number of unique BCTs used under each overarching domain as presented in the BCT Taxonomy v1 [41]

**Fig. 5:**
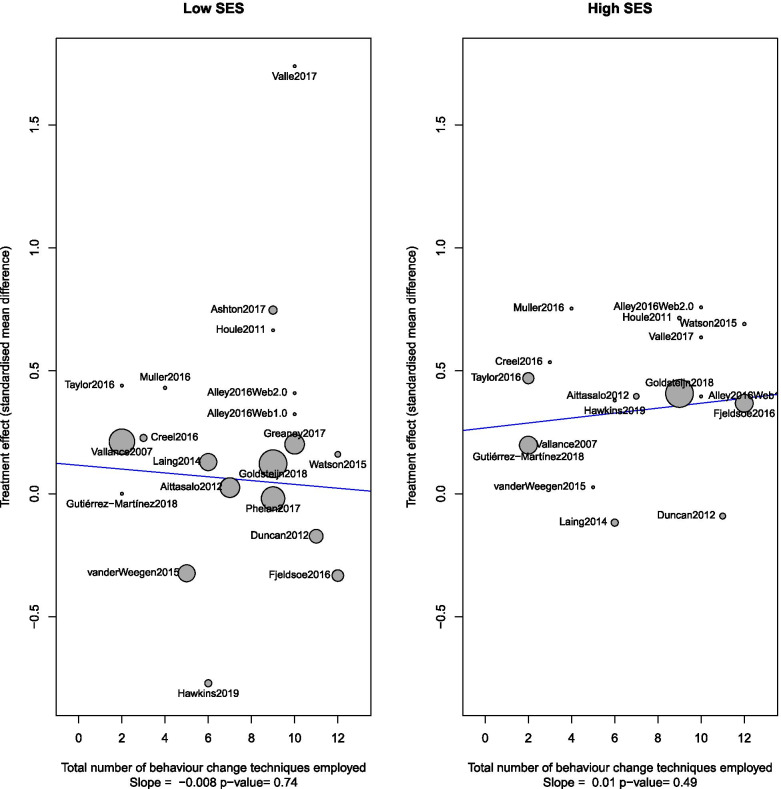
Meta-regression in low SES by the total number of BCTs for both low and high SES participants

Figure 4 displays the BCTs found in each intervention. The reviewed studies used a mean of 7 BCTs (range = 2–12). The most common BCTs were Self-monitoring of behaviour (81%), Goal setting behaviour (76%), Feedback (76%), Problem-solving (52%), Action planning (52%), Information about health consequences (48%), Behaviour goal review (43%) and Social support (43%). Post-hoc meta-regression of the number of BCTs employed by each study revealed no statistically significant trend between the amount of BCTs employed for either low or high SES groups (Fig. 5). Subgroup analysis of individual BCTs with more than one constituent indicator (goals and planning, feedback and monitoring, shaping knowledge, natural consequences, comparison of behaviour, reward and threat, and antecedents) did not indicate sub-group effects.

### Risk of bias

**Fig. 6:**
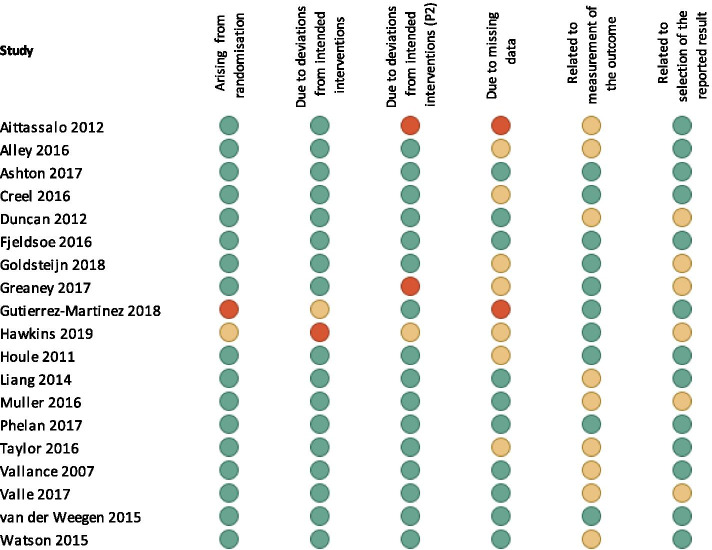
Risk of bias assessment for included studies where green = low risk of bias, amber = some concerns, and red = high risk of bias

All 19 of the included studies were assessed for risk of bias (Fig. 6). Four studies were considered low risk of bias for all categories, four had one category judged to have some concerns, six had two categories judged to have some concerns and four had at least one category judged as high risk of bias. Given the behavioural nature of the trial, blinding to allocation was not possible in any of the studies, and a distinction was made in terms of outcome measure being self-report vs. device-based, with the latter considered to incur less risk than the patient-reported former.

## Discussion

In this systematic review and meta-analysis, we provide evidence that digital behaviour change interventions aimed at increasing PA are effective for people of high SES but were not observed to be beneficial for people of low SES. In particular, our analysis of 19 studies with 20 interventions found no evidence of effect in sub-samples defined as low SES, but a statistically significant, small-to-medium effect size in high SES participants. This effect was consistently observed across SES indicators, geographical setting, the clinical status of the population, length of intervention, and PA assessment method. Most studies used self-regulatory BCTs such as self-monitoring, goal setting and feedback as the primary intervention features, but the number nor type of BCTs used in interventions were associated with the outcome in high or low SES particpants. The studies included in this review were mostly of moderate or low risk of bias.

This, to our knowledge, is the first systematic review and meta-analysis that has analysed digital interventions targeting PA behaviour according to SES. Other systematic reviews and meta-analyses have looked at digital interventions without stratifying by SES. Stockwell et al. observed a pooled standardised mean difference in PA of 0.28 across 8 RCTs of digital behaviour change interventions targeting PA in older adults [68]. Davies et al. looked at internet-delivered PA interventions in adults demonstrating a pooled effect size of 0.16 across 25 studies that used an RCT design [69]. Most recently, Laranjo and colleagues demonstrated a pooled standardised mean difference of 0.35 in their meta-analysis of 28 RCTs involving 7454 adults that underwent mobile application or activity tracker-based interventions [70]. The discrepancy in effect size observed between high and low SES in the present study may give some indication that the net benefit observed in these comparative reviews could be driven by a higher proportion of high SES research participants within the reviewed studies.

In this review, there was no indication that any methodological differences such as study duration, PA outcome measure, SES metric, country, or health status of the target population between studies had any impact on the findings. The application of digital technology varied considerably between the reviewed studies, ranging from motivational text messaging, feedback from wearable activity tracking devices, and sophisticated, multi-component, web-based interventions. The common BCTs used within these studies are akin to those found in commercial and research-based digital behaviour change tools targeting PA [71–73]. While the evidence base in favour of using digital technologies containing self-regulatory BCTs is growing [70, 74, 75], our results suggest that such interventions may be of little benefit to participants of low SES irrespective of complexity (i.e. number of BCTs) content (i.e. type of BCTs). Put another way, although access to these technologies may be improving, simply receiving interventions, even those that are efficacious in the more educated and recurrently researched higher SES populations, may not provide adequate support to those who are more deprived, less educated and/or have lower income. Consequently, more research into the BCTs that serve lower SES populations is needed.

Of course, simply receiving an intervention does not guarantee effective engagement with that intervention in a way that leads to behaviour change [76]. One important aspect we were unable to tease out from the reviewed studies is whether the dose of intervention received and the utilisation of key intervention features or BCTs were equal between high and low SES participants. Future studies deploying digital interventions for promoting PA would do well to monitor and report meaningful usage and engagement to see if this is equivalent between the low and high SES participants [77]. Additionally, people of low SES may, in general, tend to use the internet less for health information and have a lower eHealth literacy, i.e. people’s capability to use information and communication technology to improve their health, which may impact intervention engagement [30, 34, 78]. Levels of eHealth literacy are positively associated with lifestyle behaviour [79, 80]. In the context of digital PA interventions, eHealth literacy might translate as the users’ ability to navigate the technological devices themselves, understand the information received from the educational components, and appropriately apply the self-regulatory BCTs that are advocated. Incorporating intervention components that identify low eHealth literacy and boost it as a preliminary objective prior to implementing behavioural support may be one way of making these interventions more equitably beneficial.

Another possible explanation might be that the antecedents of PA may vary between people of low and high SES [81]. Pertinent frameworks of behaviour suggest that individuals need to have the capability, opportunity and motivation to be able to make changes [82]. Compared to those of low SES, people of higher SES may elicit more opportunities to act upon intervention advice or feedback through more free time, the ability to prioritise lifestyle behaviour and more resources, as well as a more supportive social and physical environment that facilitates increases in PA [83]. A range of behavioural theories was used to inform the interventions used in the reviewed studies, but it is unclear if the application of these theories was tailored in any way to meet the needs of study participants with varying SES, demographics and circumstances. As the importance of personalisation of digital behaviour change tools is increasingly recognised and tailored interventions are being implemented [76, 84, 85], ensuring that contextual factors related to SES that may influence behaviour are catered for would be a useful direction for further intervention research.

In light of the findings of this review and the acceleration towards a digital world (escalated by the COVID-19 pandemic), there is an urgent need to investigate whether digital behaviour change interventions are widening rather than reducing inequalities [86]. Our review looks at the equivalence of effect on PA behaviours, but it would not be unreasonable to assume that similar findings would be observed in research targeting other health or behavioural outcomes. Investigating whether these technologies can benefit people of low-SES, and how to improve their efficacy for this sub-population who are invariably the most in need of lifestyle support, should be a public health priority. Inevitably, developers of commercial technology for supporting PA behaviour may not prioritise lower SES segments should the goal be to maximise revenue, so the onus will likely be on researchers and public health advocates to address the discrepancy in the effectiveness of digital interventions between SES groups.

Investing in research and development for technologies that explicitly support PA among low SES populations could be a valuable public health strategy given the potential for maximising reach in populations who disproportionately utilise healthcare resources. There are certainly ways that the research community could augment progress in this area: by better reporting the SES component of their sample across multiple indices and making a concerted effort to recruit people of lower SES to their research trials so that more extensive evaluation of this sub-population can be conducted. When developing digital interventions targeting PA or other lifestyle behaviours, researchers should adopt a person-centred approach [87, 88] that encourages the use of guiding principles to ensure that the design features meet the needs and context for all individuals across the SES spectrum. Similarly, creating digital resources using participatory research which targets low SES users will help to ensure that the most pertinent BCTs and features are used in a way that will enhance engagement and the likelihood of behaviour change [89].

The key strengths of this systematic review are the comprehensive literature searching, screening, data extraction and risk of bias assessments, as well as the retrieval and analysis of raw individual level or summary data from studies regarding the SES measures from exclusively RCTs. We also observed low statistical heterogeneity (I^2^) scores in our meta-analyses, indicating a robust analytical approach when determining our pooled effect sizes.

Conversely, a limitation of this study is the high methodological heterogeneity of included studies. The variability in the SES metric, which used different constructs on varying ordinal or continuous scales, made standardising a ‘low SES’ definition or threshold across studies challenging. While every effort was made to take a systematic, time-referenced approach, it must be acknowledged that the grouping of low SES participants may not be equivalent from one study to the next. Our broad definitions of digital health and PA meant that intervention characteristics and outcome measures were variable across studies with some including more digital or non-digital components than others, which makes unpacking the specific mechanisms that drive the findings difficult in the present study. Similarly, the control groups included in the review differed from one study to the next, which could explain the lack of observed effect in low SES groups, although it should be acknowledged that this would not explain the discrepancy in effects observed within a study between low and high SES participants. A further limitation is the searching of literature, which, by solely targeting research databases and articles written in the English language, may not have included all available research on this topic. Similarly, there were eligible articles for which study authors were unable to provide the necessary stratified data and further titles that will have been published following the analysis and publication of the present systematic review.

## Conclusions

In this study, we demonstrate that, at present, digital interventions targeting PA are not equivalently effective for people of low and high SES. Specifically, there is very little evidence that digital PA interventions have any efficacy for people of low SES, but moderate efficacy for those of high SES, both between and within studies. Increasing access to information communication and wearable technology amongst even the most vulnerable people has led to digital interventions being championed as a tool for reducing inequalities in health promotion. This study suggests that in a PA context the opposite is true, that is, people who would benefit the most from these interventions are being left behind. We recommend that future development of digital interventions aimed at improving PA must make more effort to meet the needs of low SES people within the target population.

## Supplementary Information

**Additional file 1.**

**Additional file 2.**

**Additional file 3.**

## Data Availability

All data and code to replicate our analysis are freely available at https://github.com/MarkKelson/REPAID
