# Supplementary material for: The effectiveness of digital interventions for increasing physical activity in individuals of low socioeconomic status: a systematic review and meta-analysis
Source: Int J Behav Nutr Phys Act. 2021 Nov 9;18:148. doi: 10.1186/s12966-021-01218-4 (PMC8576797; doi:10.1186/s12966-021-01218-4)
Supplement: Supplementary file 1 — Additional file 1. [file 12966_2021_1218_MOESM1_ESM.docx]

Search terms used:

1. exp Mobile Applications/

2. ((mobile* or smartphone* or phone* or iphone* or cellphone* or web-based or website* or online or internet or tablet* or ipad* or android or windows or techn*) adj3 (app or apps or application* or platform* or program* or interface*)).tw.

3. mhealth.tw.

4. m-health.tw.

5. ehealth.tw.

6. e-health.tw.

7. pedometer*.tw.

8. acceleromet*.tw.

9. tele-acceleromet*.tw.

10. etechnolog*.tw.

11. e-technolog*.tw.

12. fitness band*.tw.

13. exp Fitness Trackers/

14. ((fitness or activ*) adj3 track*).tw.

15. (wearable adj3 (monitor* or sensor*)).tw.

16. tracker*.tw.

17. tracking tech*.tw.

18. physical activity monitor*.tw.

19. physical activity sensor*.tw.

20. wearable proximity sensor*.tw.

21. wearable proximity monitor*.tw.

22. or/1-21

23. exp Exercise/

24. exercis*.tw.

25. (physical* adj3 activ*).tw.

26. (physical* adj3 exert*).tw.

27. (health* adj3 lifestyle*).tw.

28. ((inactive* or in-activ*) adj3 lifestyle*).tw.

29. workout.tw.

30. exp Walking/

31. walk*.tw.

32. step*.tw.

33. sport*.tw.

34. MVPA.tw.

35. IPAQ.tw.

36. LTPA.tw.

37. metabolic equivalent.tw.

38. sedentary energy expenditure.tw.

39. PAEE.tw.

40. exp Sedentary Lifestyle/

41. sedentary.tw.

42. PAL.tw.

43. (MET minute or MET-minute or MET hour or MET-hour).tw.

44. or/23-43

45. exp Social Class/

46. (socioeconomic status* or socio-economic status* or socio economic status*).tw.

47. SES.tw.

48. social class*.tw.

49. (socioeconomic class* or socio-economic class* or socio economic class*).tw.

50. index of multiple deprivation or IMD.tw.

51. exp Socioeconomic Factors/

52. exp Poverty/

53. poverty.tw.

54. townsend.tw.

55. (deprivation or deprived or disadvantaged).tw.

56. exp Employment/

57. (employed or employment).tw.

58. (education* adj2 attainment*).tw.

59. exp Income/

60. income*.tw.

61. inequalit*.tw.

62. or/45-61

63. 22 and 43 and 62

64. Randomized Controlled Trials as Topic/

65. randomized controlled trial/

66. Random Allocation/

67. Double Blind Method/

68. Single Blind Method/

69. clinical trial/

70. clinical trial, phase i.pt.

71. clinical trial, phase ii.pt.

72. clinical trial, phase iii.pt.

73. clinical trial, phase iv.pt.

74. controlled clinical trial.pt.

75. randomi?ed controlled trial.pt.

76. multicenter study.pt.

77. clinical trial.pt.

78. exp Clinical Trials as topic/

79. or/64-78

80. (clinical adj trial$).tw.

81. ((singl$ or doubl$ or treb$ or tripl$) adj (blind$3 or mask$3)).tw.

82. PLACEBOS/

83. placebo$.tw.

84. randomly allocated.tw.

85. (allocated adj2 random$).tw.

86. or/80-85

87. 79 or 86

88. case report.tw.

89. letter/

90. historical article/

91. or/88-90

92. 87 not 91

93. 63 and 92

**APPENDIX 2**

| **Data Extraction Form** | | |
| --- | --- | --- |
| **A systematic review on the effectiveness of mobile/web-based apps to increase physical activity in individuals from a low socioeconomic background.** | | |
| **General information** |  |  |
| Study Citation and author email: | Data extracted by: First reviewer | Data checked by: Second reviewer |
|  | Date | Date: |
| Study aims: |  |  |
| Study Design: |  |  |
| **Include/ Exclude** |  |  |
| **Reason for exclusion:** |  |  |
|  |  |  |
|  |  |  |
| **Participants and setting** |  |  |
| ***Population: Intervention group*** |  |  |
| *Age* |  |  |
| *Sex* |  |  |
| *SES* |  |  |
| *Pathology* |  |  |
| *Pregnancy* |  |  |
| ***Population: Control group*** |  |  |
| *Age* |  |  |
| *Gender* |  |  |
| *SES: definition, determinants* |  |  |
| *Pathology* |  |  |
| *Pregnancy* |  |  |
| ***Setting:*** |  |  |
| *Context* |  |  |
| *Location* |  |  |
| *Country* |  |  |
| ***Other relevant information*** |  |  |
| ***Inclusion criteria:*** |  |  |
| *Physical activity measured* |  |  |
| *Apps used as primary intervention* |  |  |
| *RCT* |  |  |
| *Results reported by SES* |  |  |
|  |  |  |
| ***Exclusion criteria:*** |  |  |
| *Any pharmacological component used alongside the apps* |  |  |
| *An exclusive focus given on rare diseases or conditions* |  |  |
| *Results rported not in English* |  |  |
|  |  |  |
| **Intervention** |  |  |
| Description (type, model) |  |  |
| Duration |  |  |
| Frequency |  |  |
| Follow up |  |  |
| BCTs used |  |  |
| Any other information |  |  |
| **Comparator** |  |  |
| Description (what was used by the control group) |  |  |
|  |  |  |
| **Outcome/ Evaluation** |  |  |
| ***Primary outcome measures:*** |  |  |
| *Primary outcome* |  |  |
| *Results* |  |  |
| *Statistical method(s) used* |  |  |
| *Effect size* |  |  |
| ***Other outcomes measures: (if relevant)*** |  |  |
| *Other outcomes* |  |  |
| *Results* |  |  |
| *Statistical method(s) used* |  |  |
| *Effect size* |  |  |
| **Other** |  |  |
|  |  |  |
| Ethical approval: |  |  |
| Limitations (author): |  |  |
| Limitations (reviewer): |  |  |
| Funding sources: |  |  |
| Conflicts of interest: |  |  |
