# Supplementary material for: The effectiveness of digital interventions for increasing physical activity in individuals of low socioeconomic status: a systematic review and meta-analysis
Source: Int J Behav Nutr Phys Act. 2021 Nov 9;18:148. doi: 10.1186/s12966-021-01218-4 (PMC8576797; doi:10.1186/s12966-021-01218-4)
Supplement: Supplementary file 2 — Additional file 2. [file 12966_2021_1218_MOESM2_ESM.pdf]

Supplementary material

Appendix A

Pre-specified sensitivity analyses relating to objective 1 (effectiveness in low SES groups)

Low SES subgroup analysis comparing apps with a sole PA focus versus those without

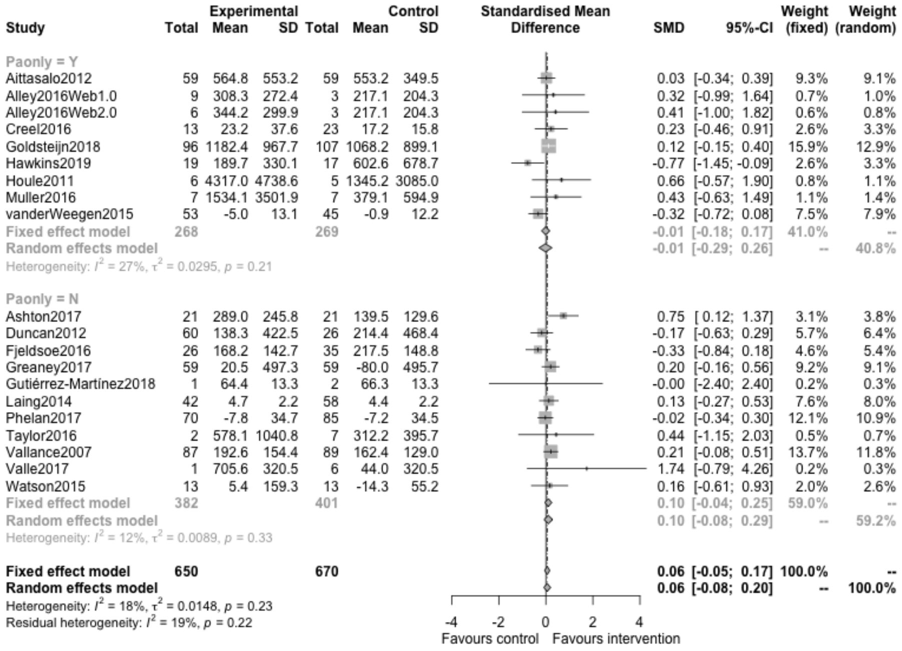

Figure 1: Low SES participants in studies split by whether the app had a sole PA focus or not

Low SES subgroup analysis comparing between countries

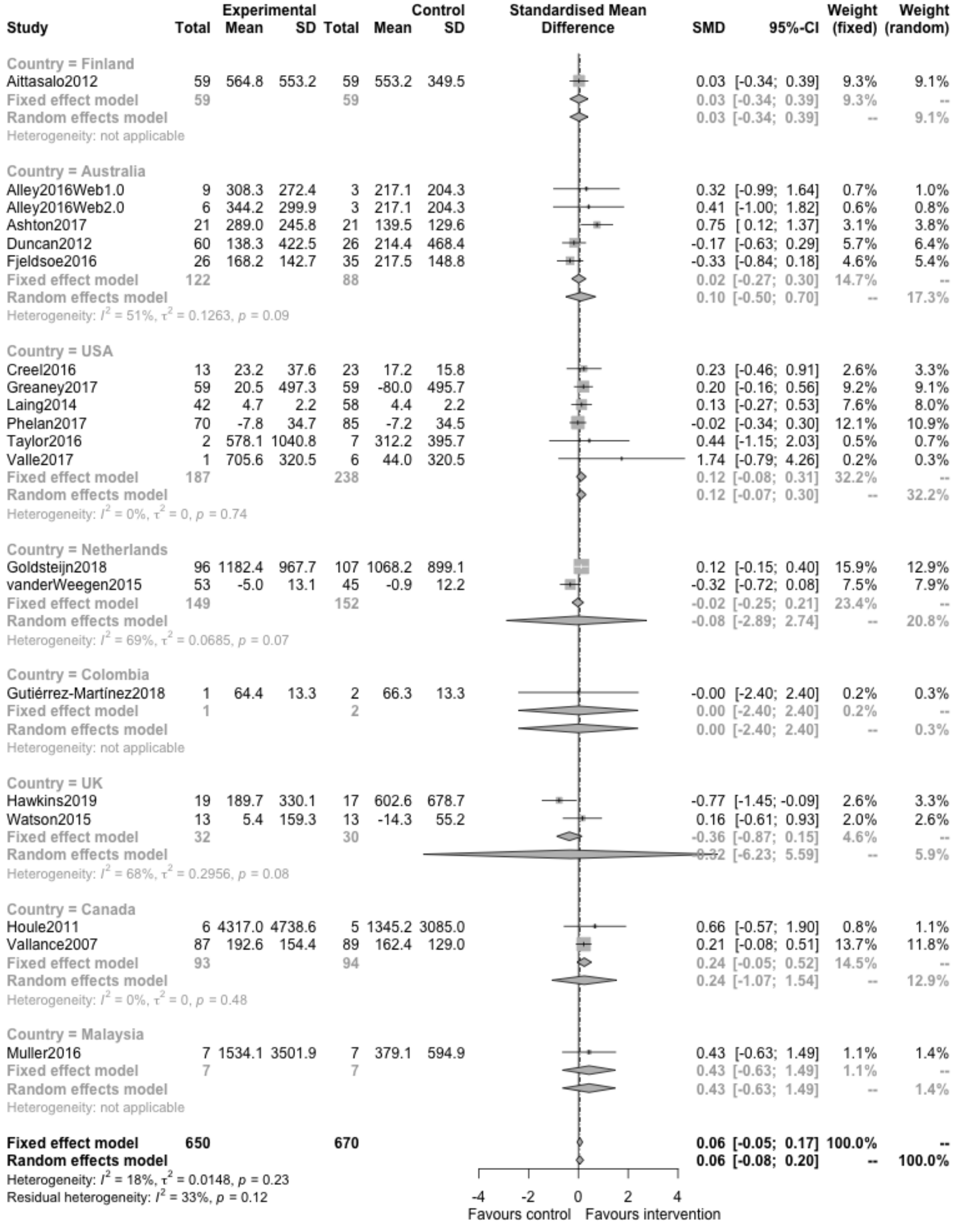

Figure 2: Low SES participants in studies split by country

Low SES subgroup analysis by outcome

There were too few studies for each outcome type to subgroup meaningfully.

Low SES subgroup analysis by high risk of bias

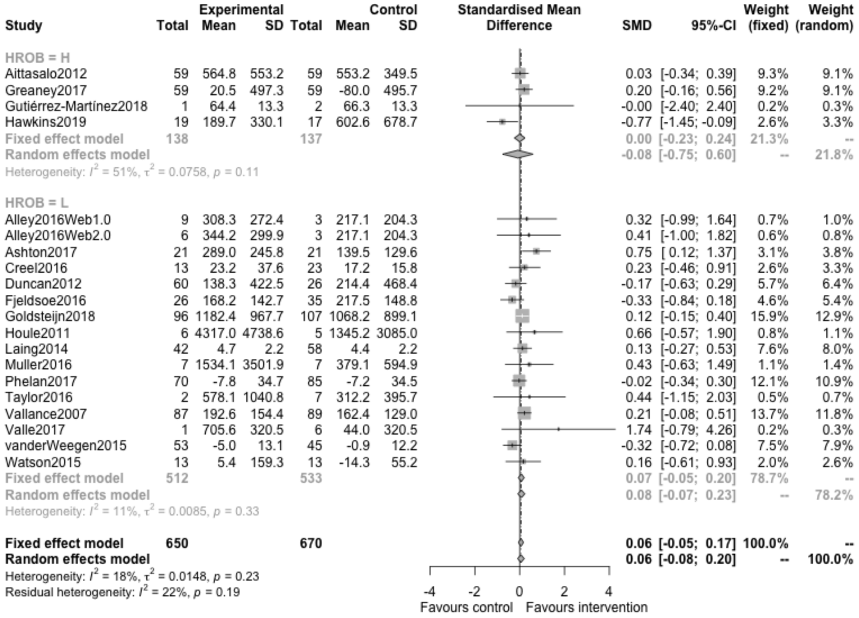

Figure 3: Low SES participants in studies split by whether the studies were at high risk of bias or not

Low SES subgroup analysis by age groups

Age group categorisation and reporting did not allow combining into groups for sub-group analysis.

Low SES subgroup analysis by healthy or general population/versus chronic disease populations

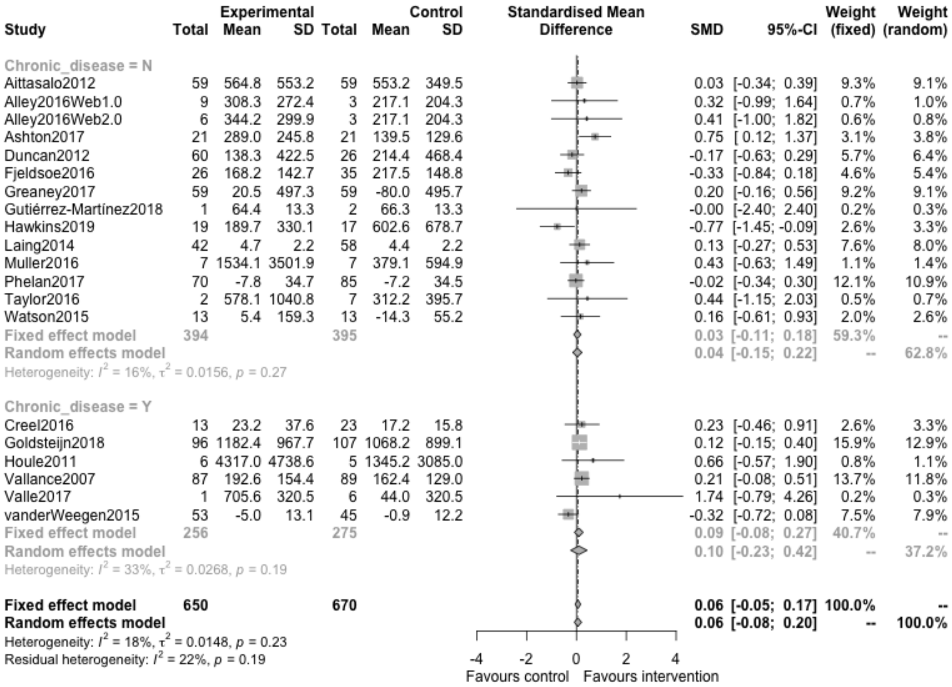

Figure 4: Low SES participants in studies split by whether the study populations where suffering fron chronic disease or not

Low SES subgroup analysis by duration of app exposure (less than 3 months, 3-6, more than 6 months)

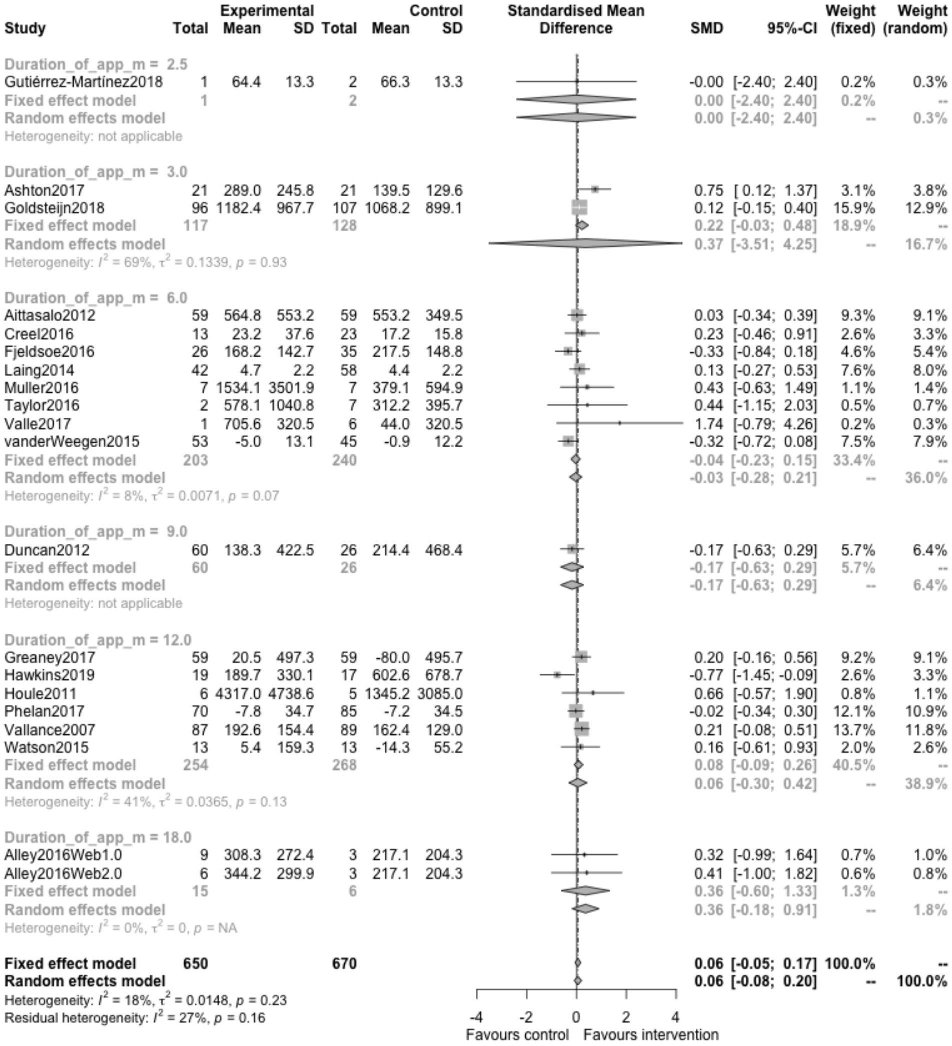

Figure 5: Low SES participants in studies split by duration of app exposure

Low SES subgroup analysis by duration of follow-up

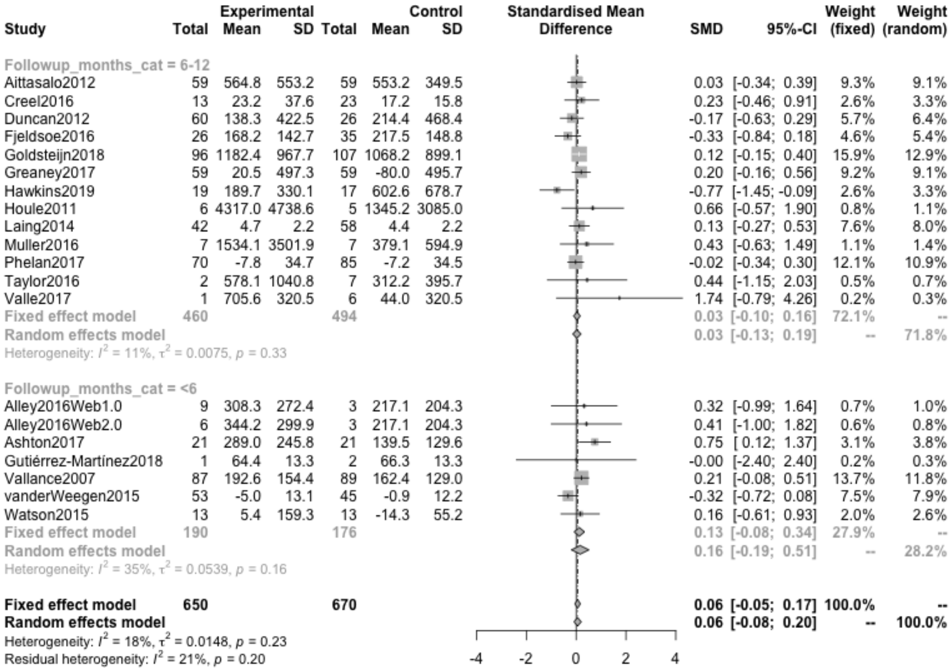

Figure 6: Low SES participants in studies split by length of follow-up

Low SES subgroup analysis by pregnancy

Not performed due to lack of studies.

Low SES Behaviour change subgroups

Low SES- Goals and planning versus not

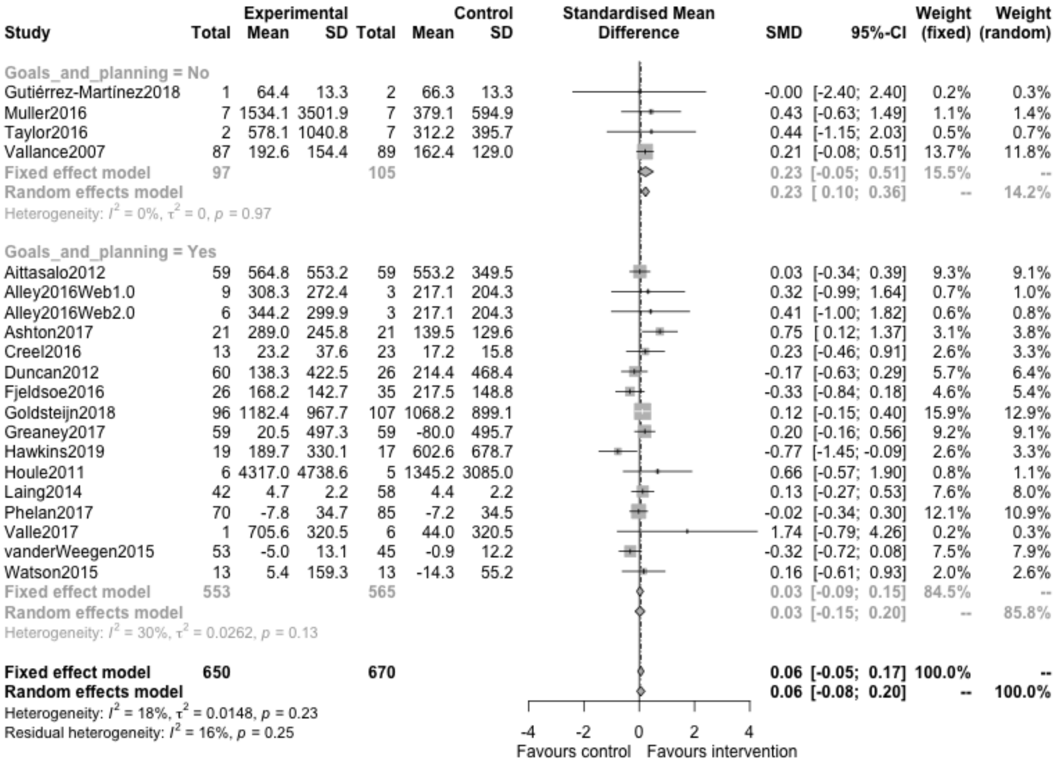

Figure 7: Low SES participants in studies split by whether they employed goals and planning as a behaviour change technique or not

Low SES- Feedback and monitoring versus not

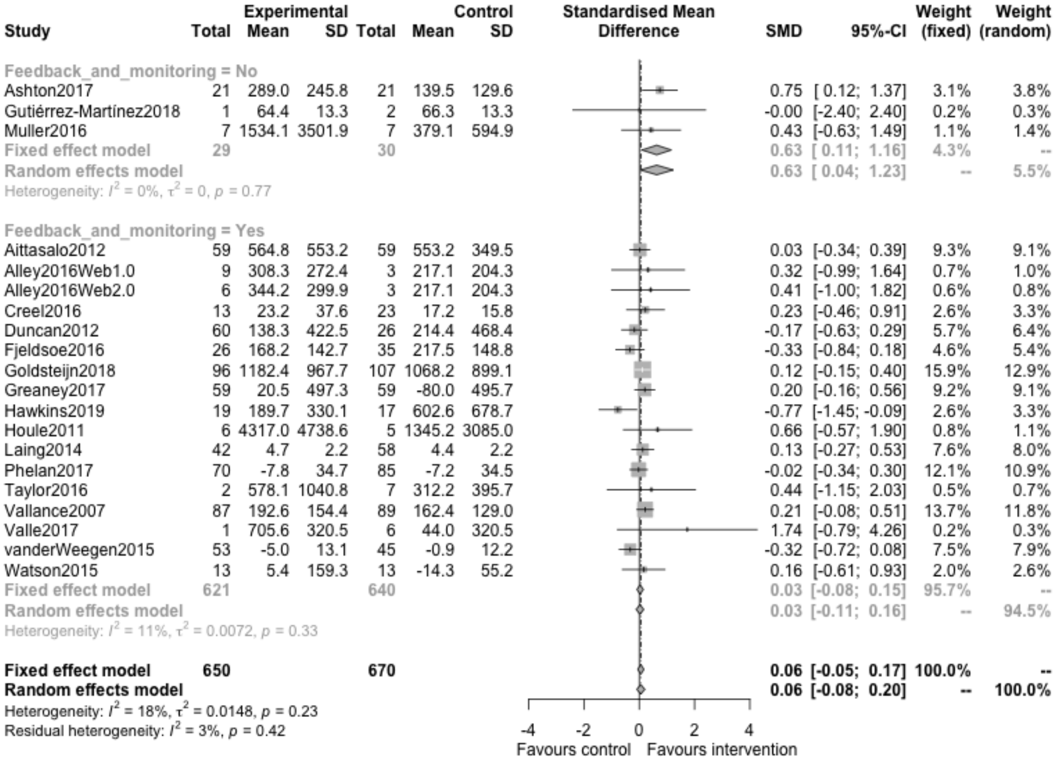

Figure 8: Low SES participants in studies split by whether they employed feedback and monitoring as a behaviour change technique or not

Low SES- Sharing knowledge versus not

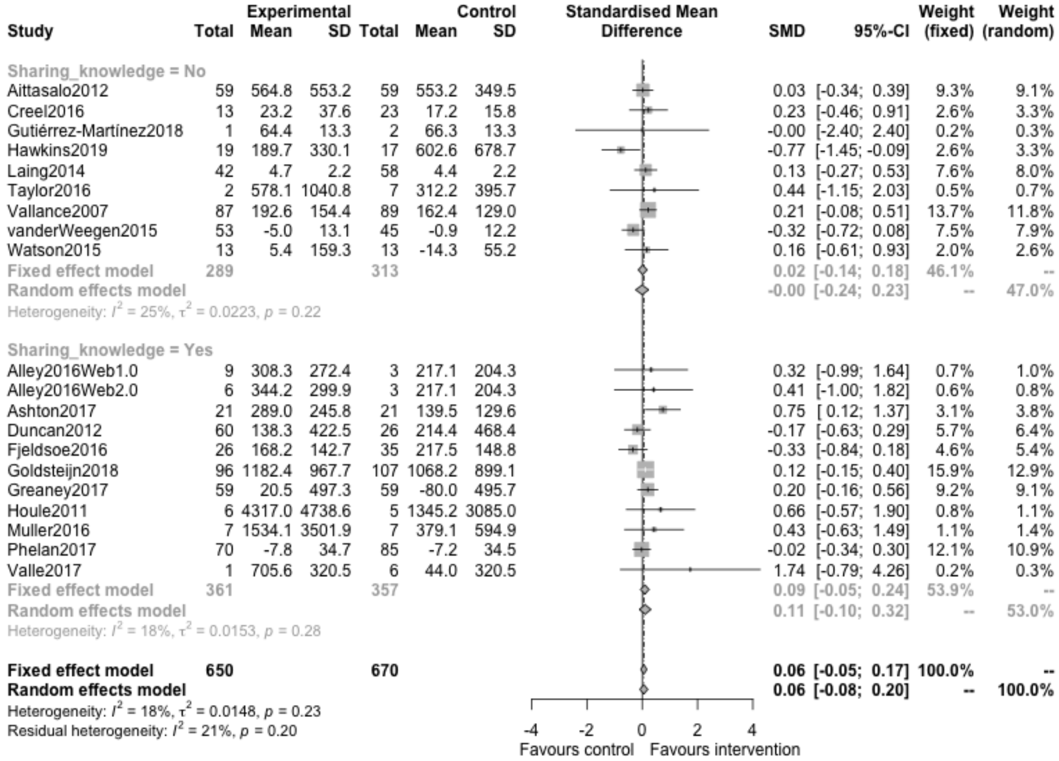

Figure 9: Low SES participants in studies split by whether they employed sharing knowledge as a behaviour change technique or not

Low SES- Natural consequences versus not

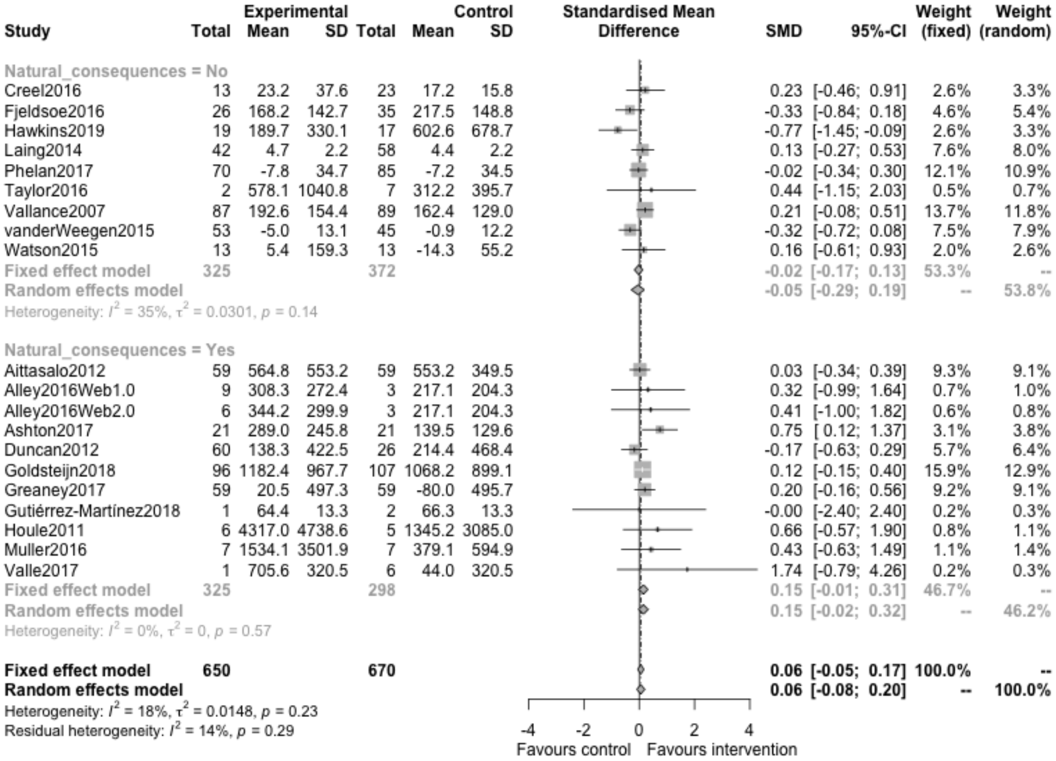

Figure 10: Low SES participants in studies split by whether they employed natural consequences as a behaviour change technique or not

Low SES- Comparison of behaviour versus not

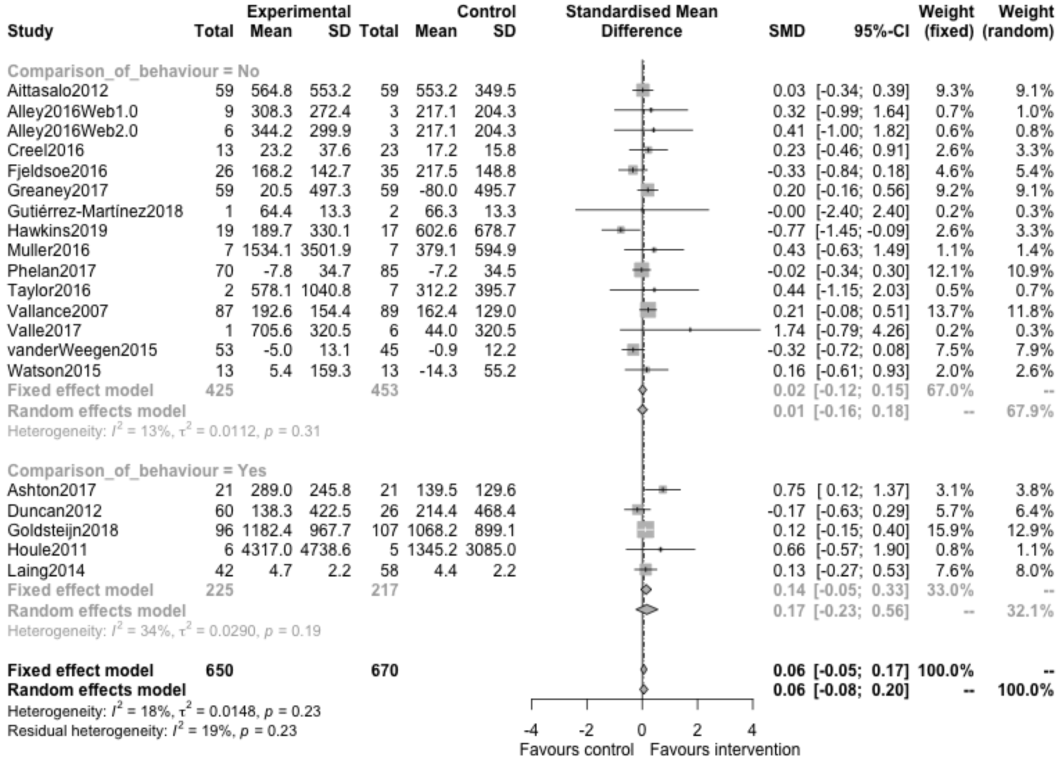

Figure 11: Low SES participants in studies split by whether they employed comparison of behaviour as a behaviour change technique or not

Low SES- Reward and threat versus not

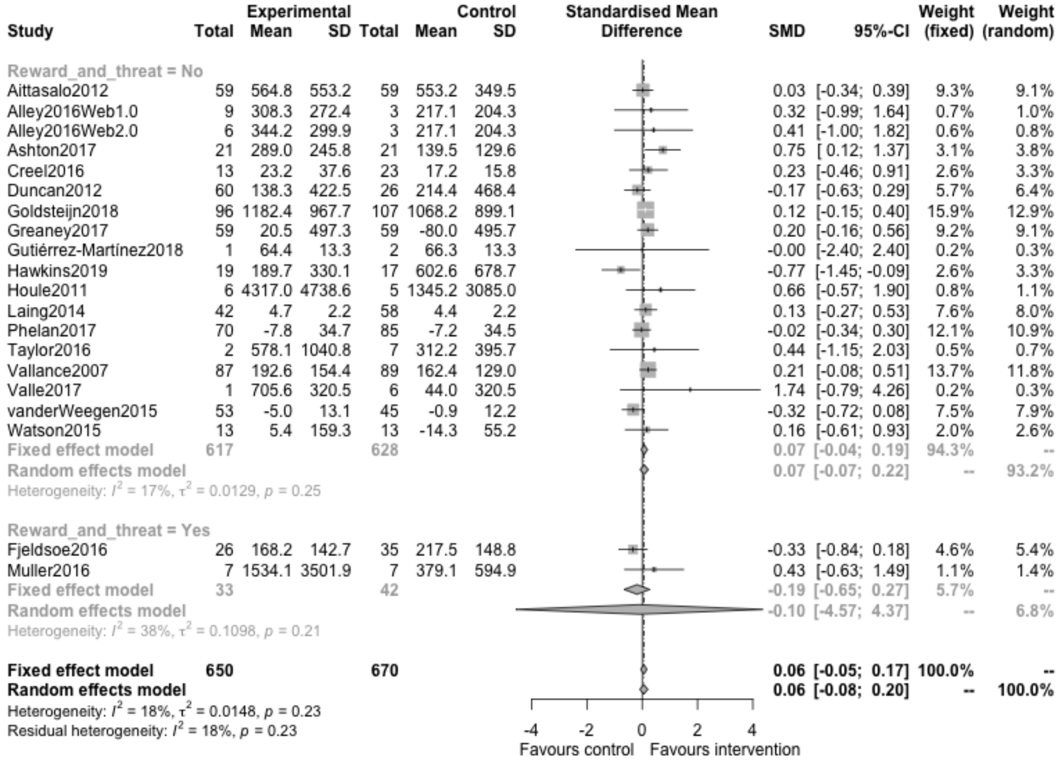

Figure 12: Low SES participants in studies split by whether they employed reward and threat as a behaviour change technique or not

Low SES- Antecedents versus not

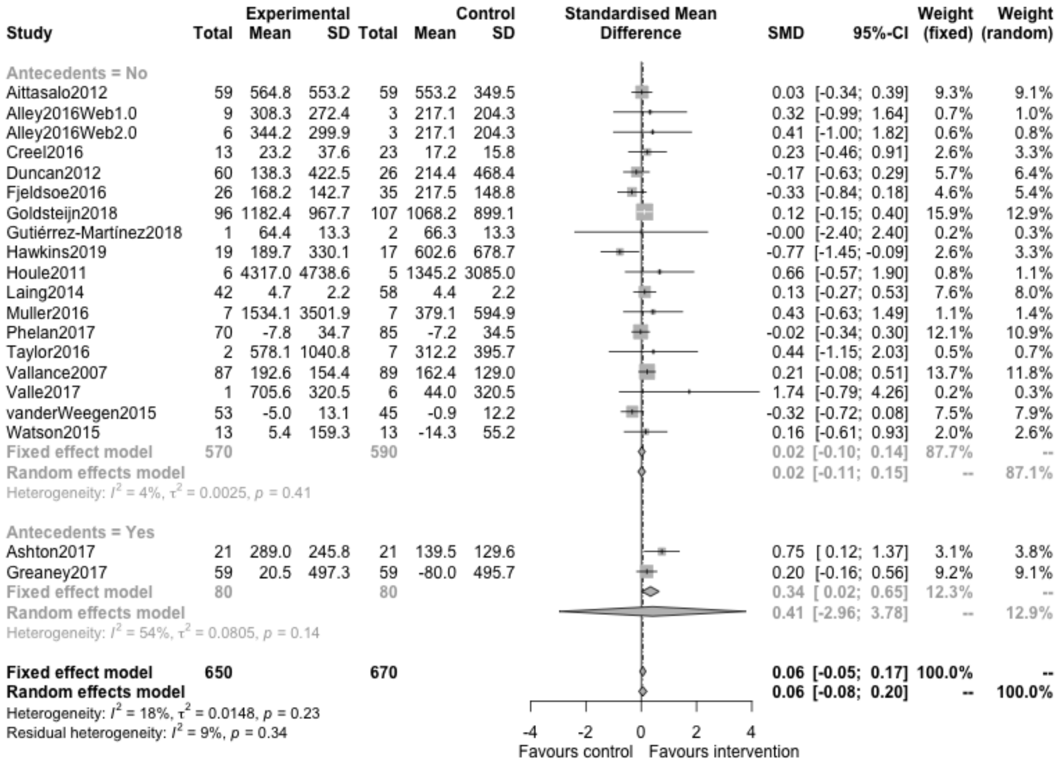

Figure 13: Low SES participants in studies split by whether they employed antecedents as a behaviour change technique or not

Appendix B

Exploration of publication bias in studies of low SES participants

There is no evidence of publication case (p-value = 0.52).

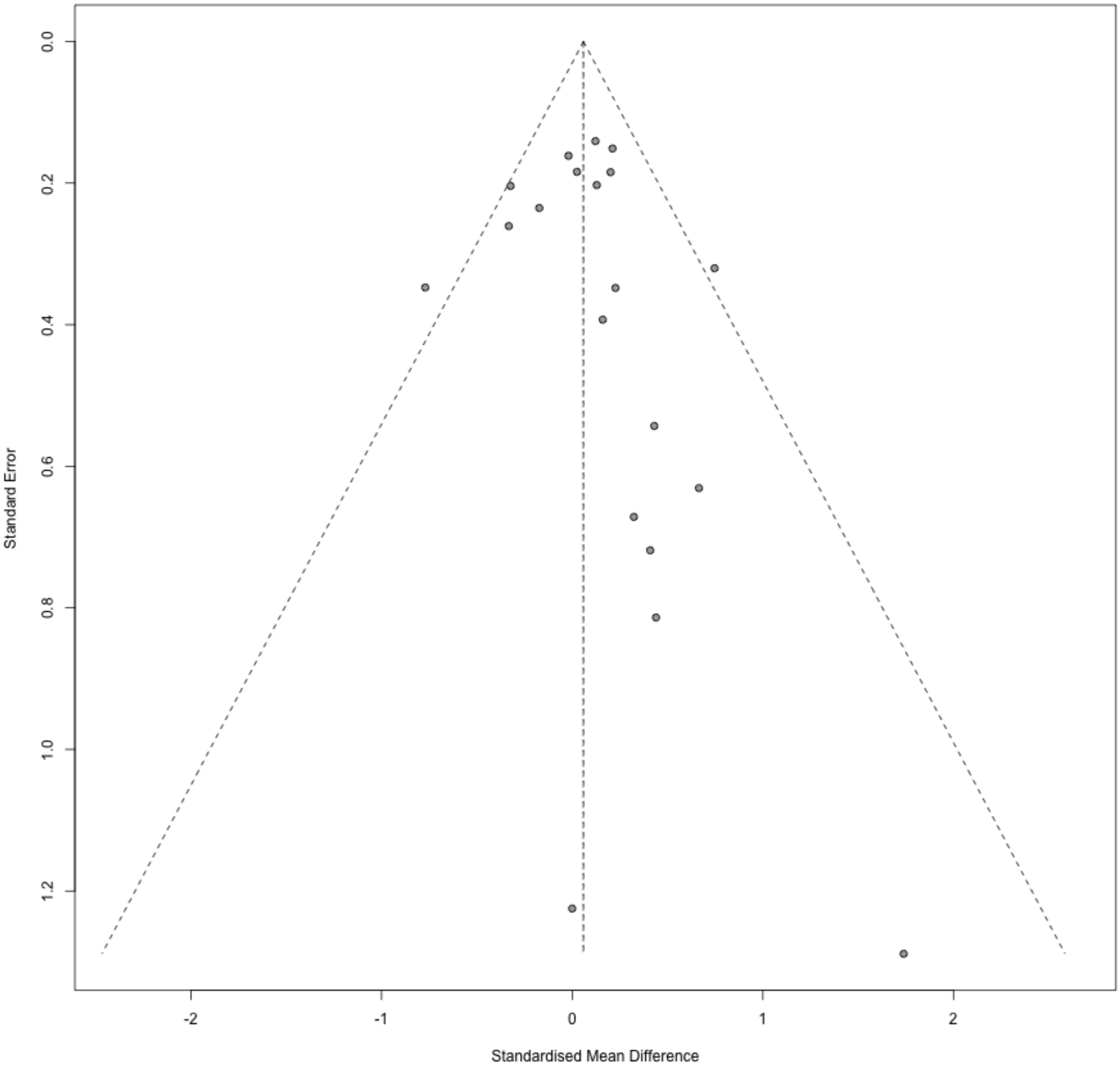

Figure 14: Funnel plot for low SES studies

Appendix C

Pre-specified sensitivity analyses relating to objective 2 (effectiveness in high SES groups)

High SES subgroup analysis comparing apps with a sole PA focus versus those without

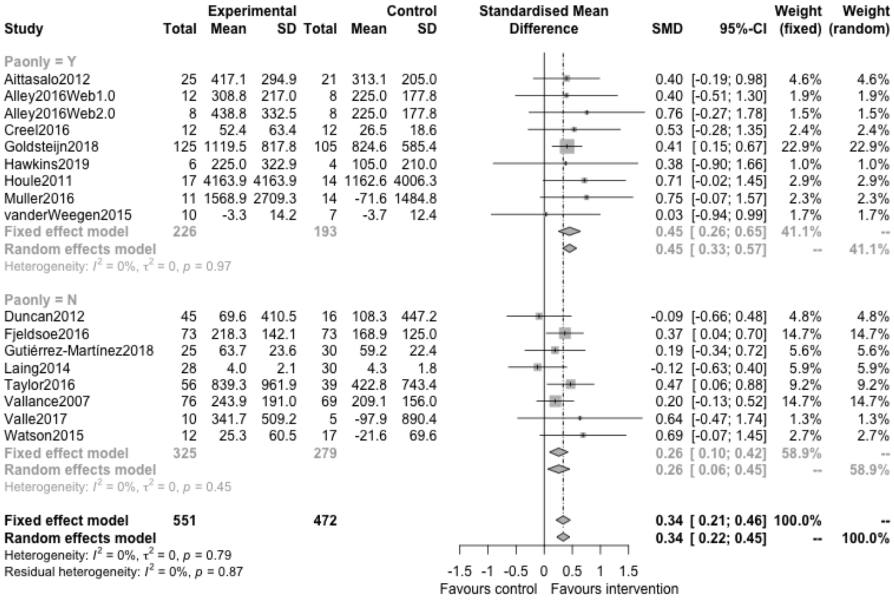

Figure 15: High SES participants in studies split by whether the app had a sole PA focus or not

High SES subgroup analysis comparing between countries

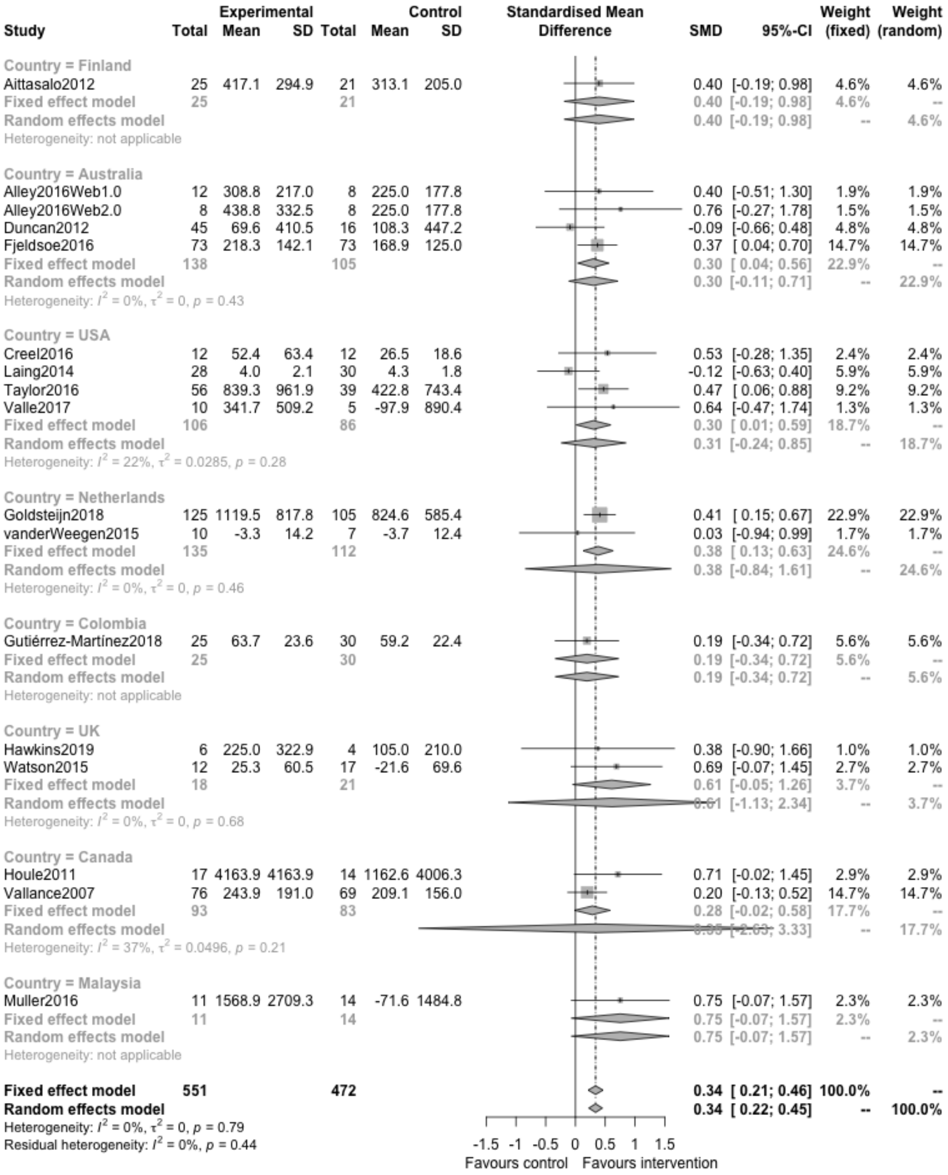

Figure 16: High SES participants in studies split by country

High SES subgroup analysis by outcome

There were too few studies for each outcome type to subgroup meaningfully.

High SES subgroup analysis by high risk of bias

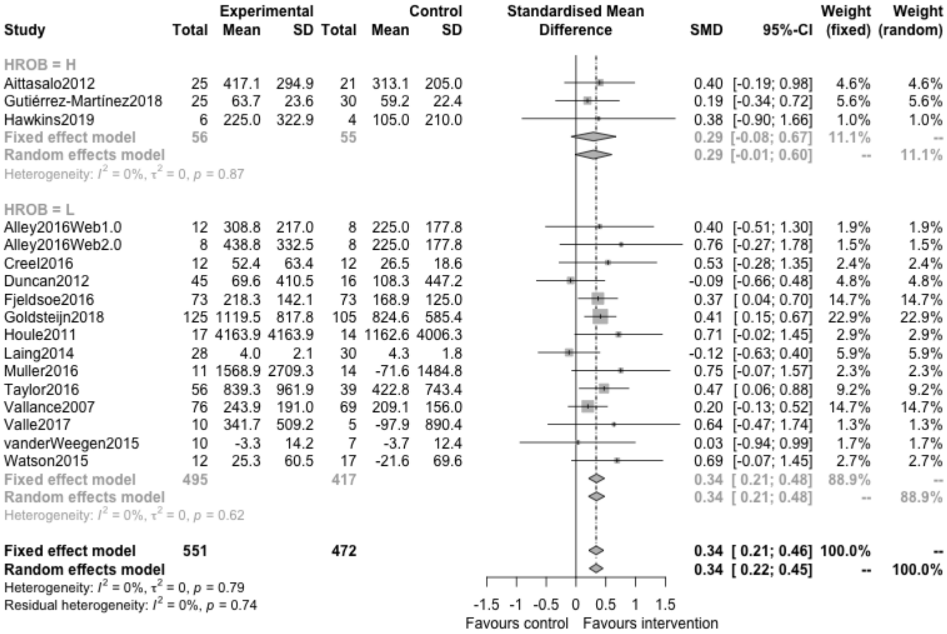

Figure 17: High SES participants in studies split by whether the studies were at high risk of bias or not

High SES subgroup analysis by age groups

Age group categorisation and reporting did not allow combining into groups for sub-group analysis.

High SES subgroup analysis by healthy or general population/versus chronic disease populations

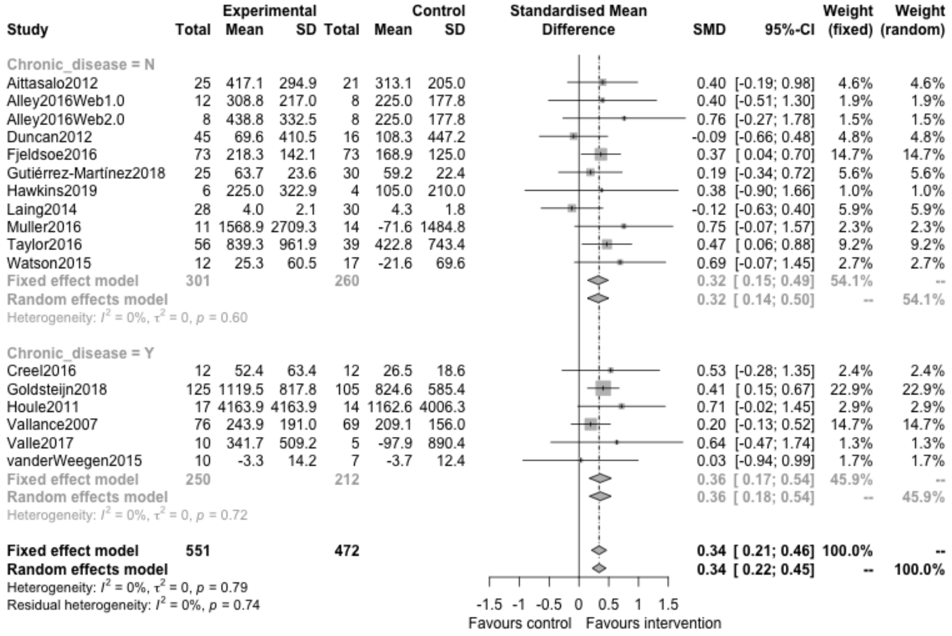

Figure 18: High SES participants in studies split by whether the study populations where suffering fron chronic disease or not

High SES subgroup analysis by duration of app exposure (less than 3 months, 3-6, more than 6 months)

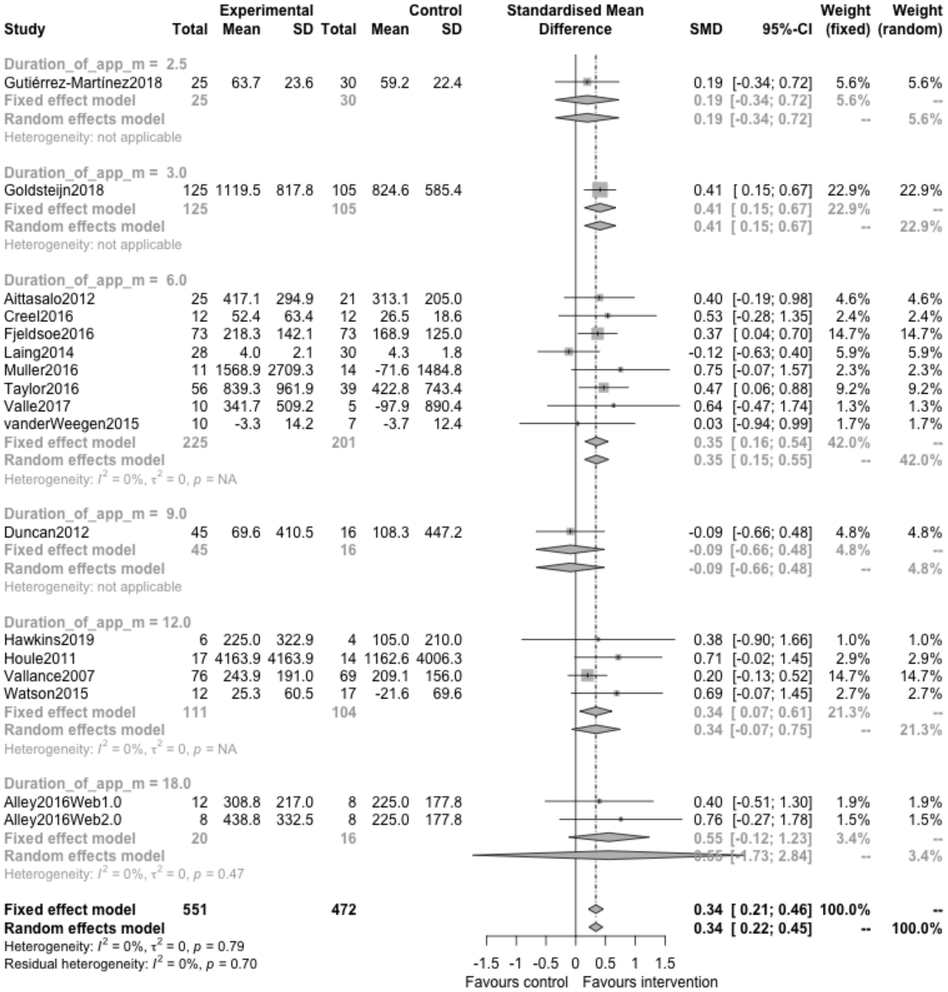

Figure 19: High SES participants in studies split by duration of app exposure

High SES subgroup analysis by duration of follow-up

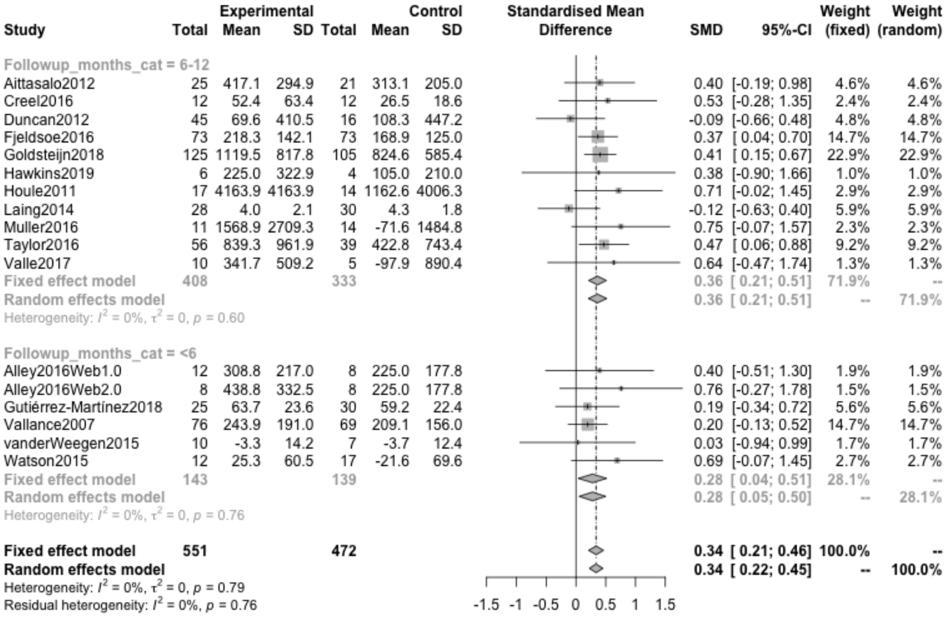

Figure 20: High SES participants in studies split by length of follow-up

High SES subgroup analysis by pregnancy

Not performed due to lack of studies.

High SES - Behaviour change subgroups

High SES- Goals and planning versus not

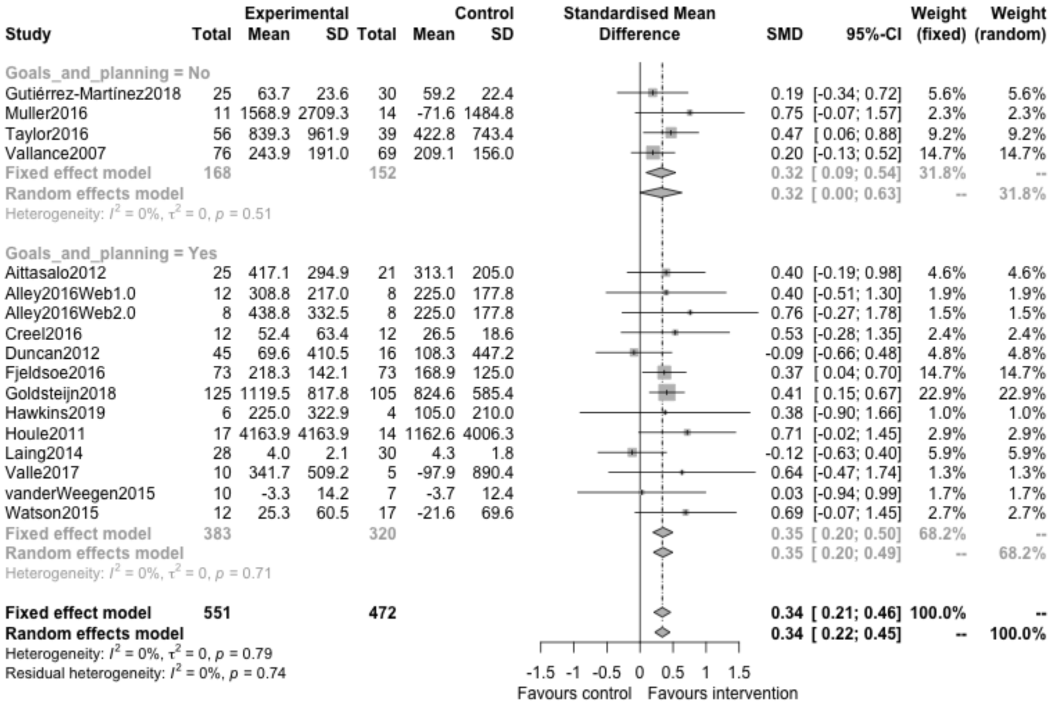

Figure 21: High SES participants in studies split by whether they employed goals and planning as a behaviour change technique or not

High SES- Feedback and monitoring versus not

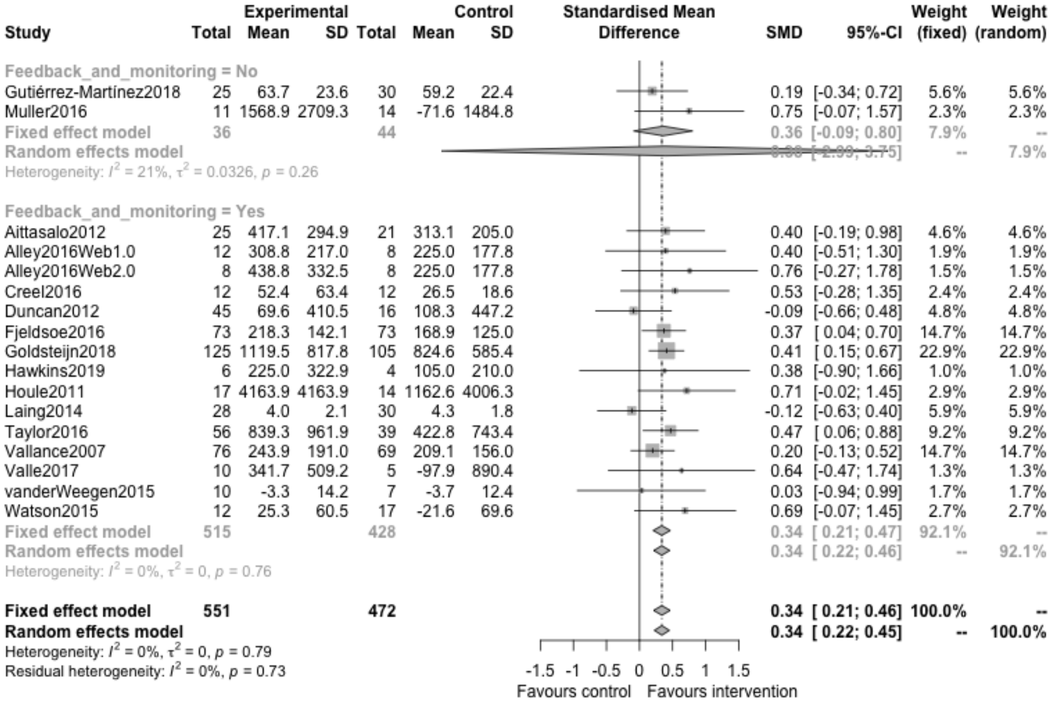

Figure 22: High SES participants in studies split by whether they employed feedback and monitoring as a behaviour change technique or not

High SES- Sharing knowledge versus not

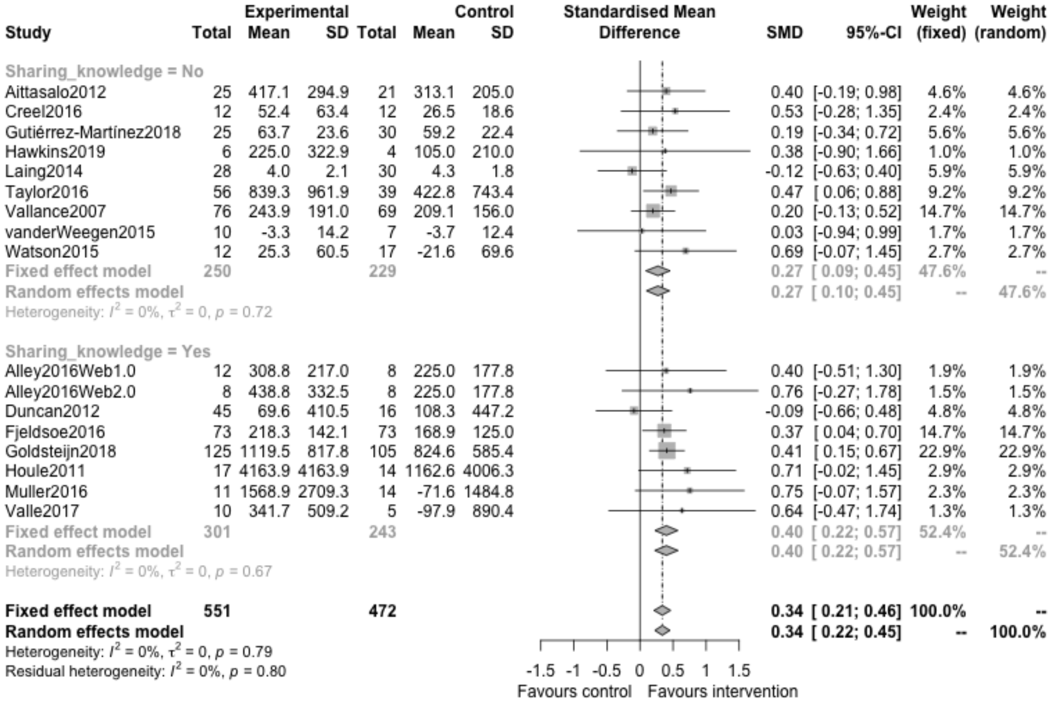

Figure 23: High SES participants in studies split by whether they employed sharing knowledge as a behaviour change technique or not

High SES- Natural consequences versus not

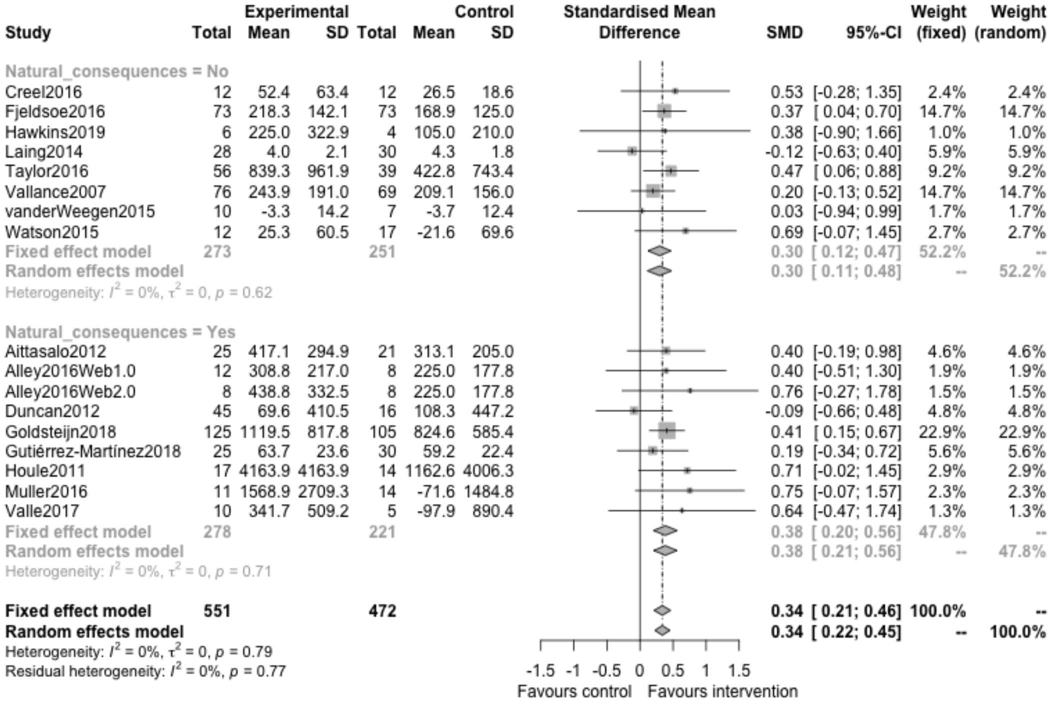

Figure 24: High SES participants in studies split by whether they employed natural consequences as a behaviour change technique or not

High SES- Comparison of behaviour versus not

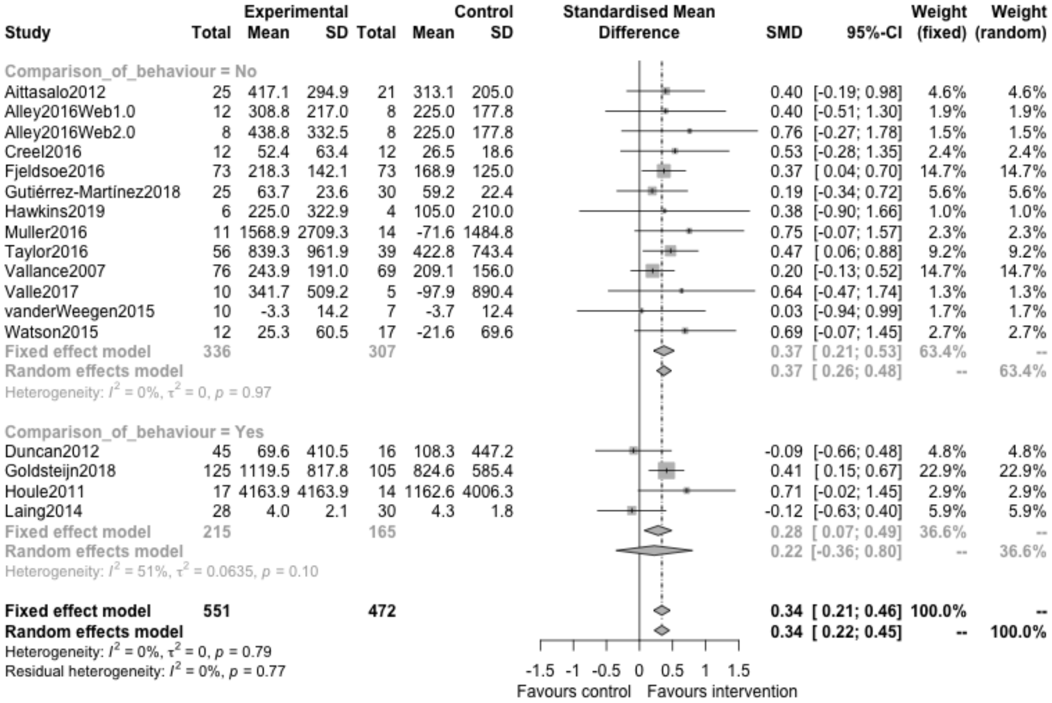

Figure 25: High SES participants in studies split by whether they employed comparison of behaviour as a behaviour change technique or not

High SES- Reward and threat versus not

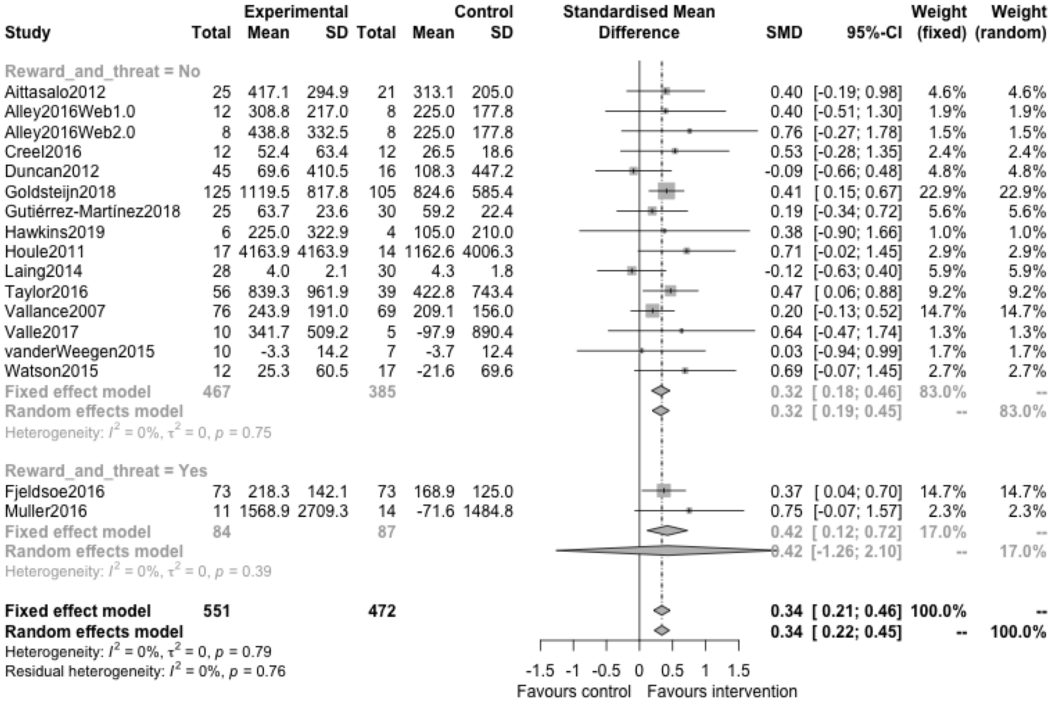

Figure 26: High SES participants in studies split by whether they employed reward and threat as a behaviour change technique or not

High SES- Antecedents versus not

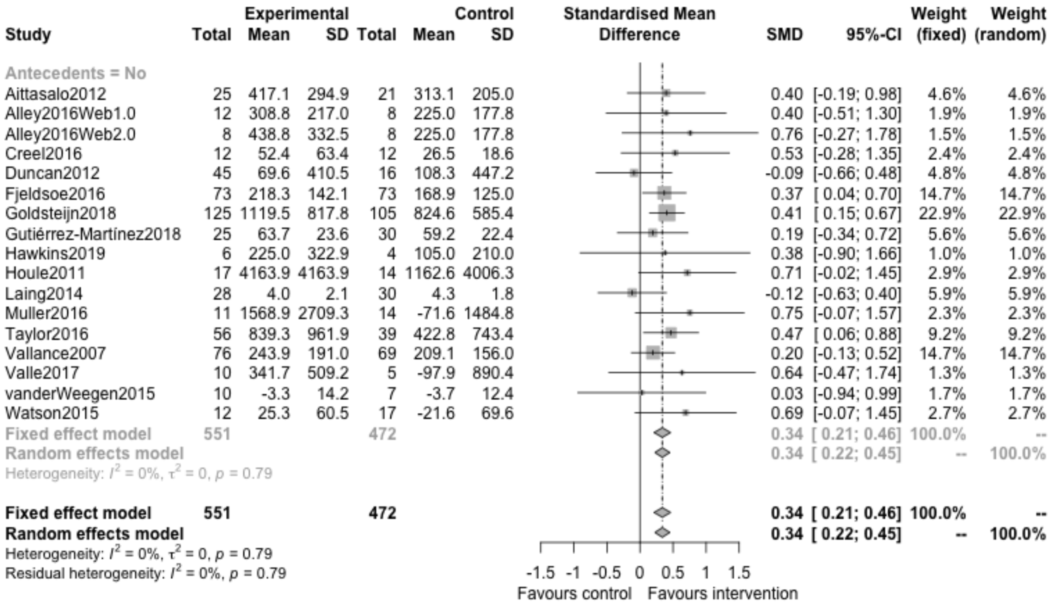

Figure 27: High SES participants in studies split by whether they employed antecedents as a behaviour change technique or not

Appendix D

Exploration of publication bias in studies of low SES participants

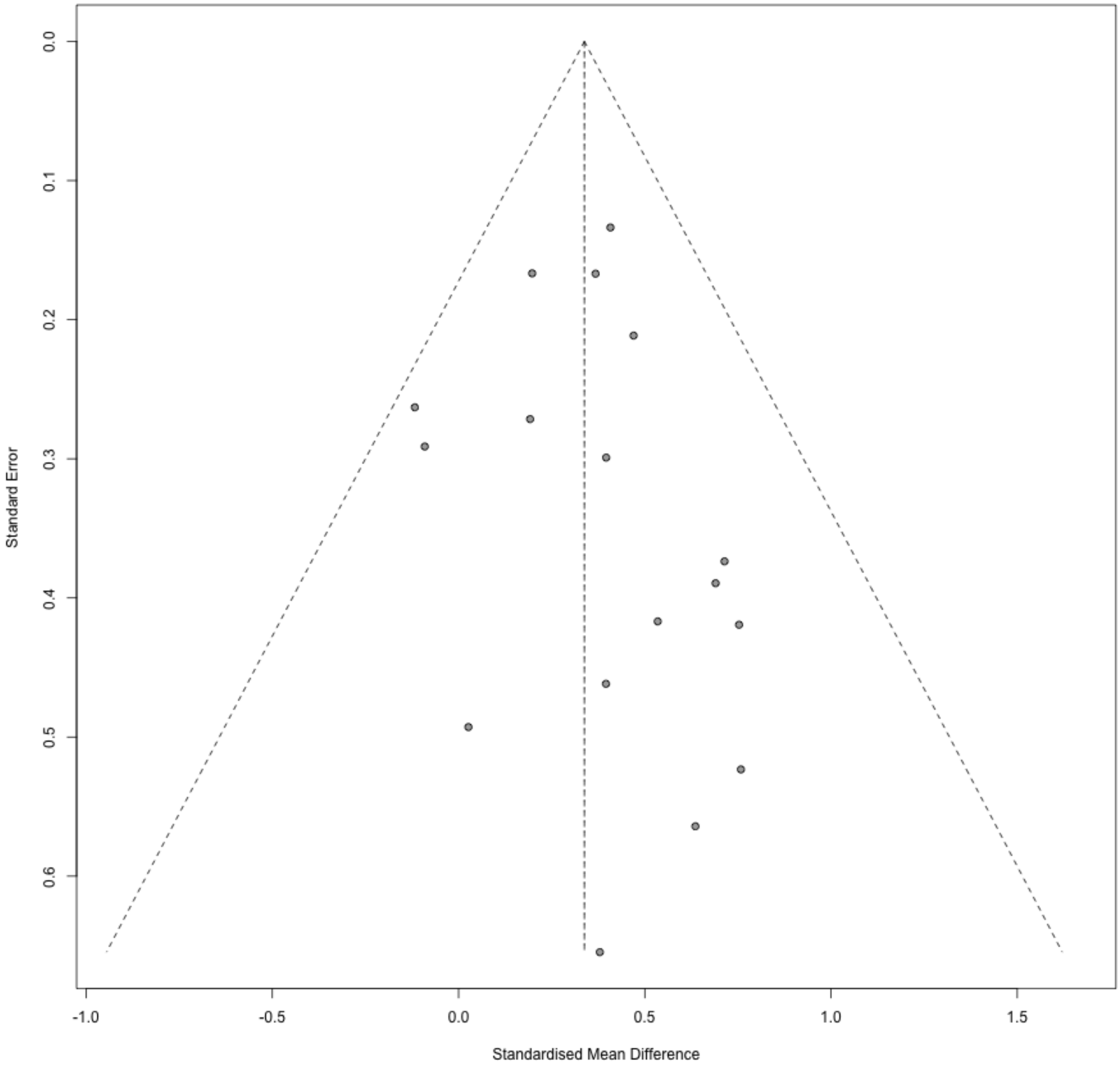

Figure 28: Funnel plot for low SES studies

There is no evidence of publication case (p-value = 0.86).

Appendix E

Post-hoc exploration of difference between objective and self-report physical activity

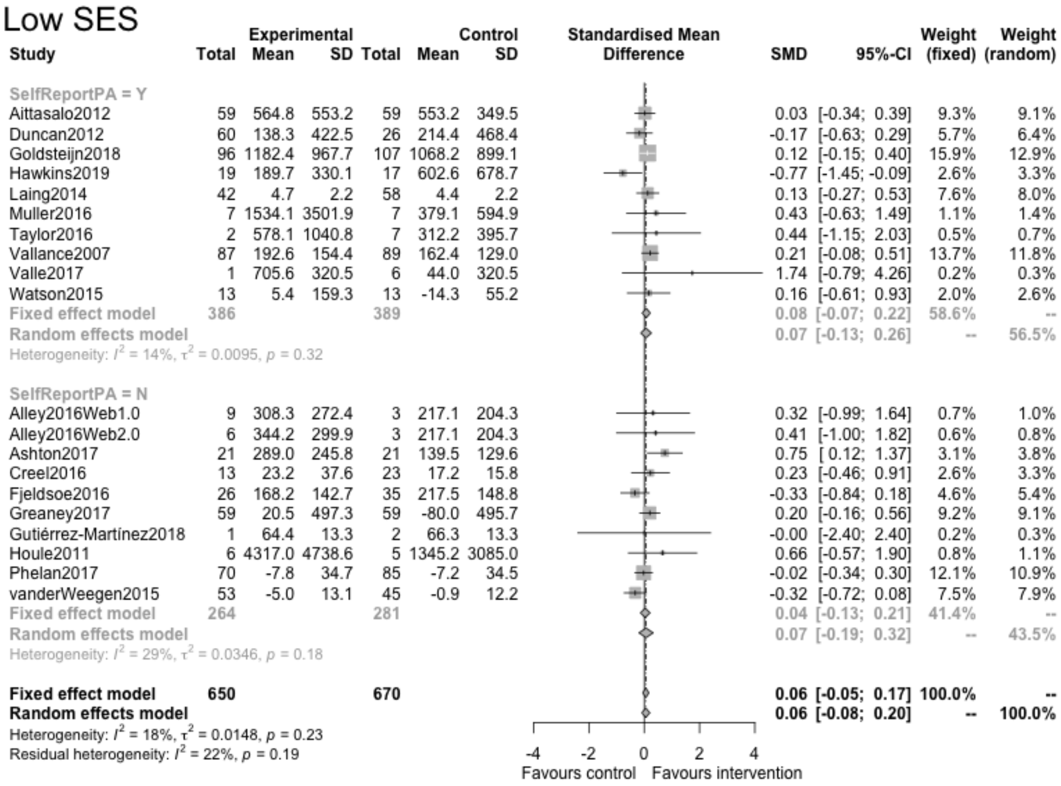

Figure 29: Post-hoc sensitivity analysis exploring differences by self-report versus objectively measured physical activity in low SES groups

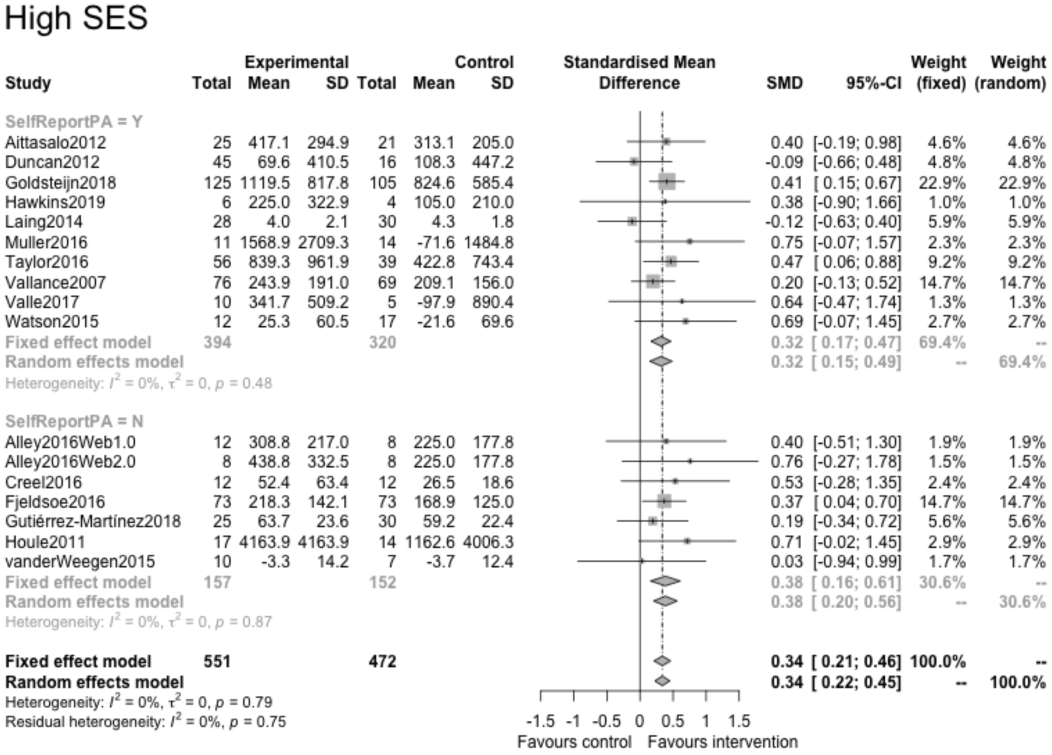

Figure 30: Post-hoc sensitivity analysis exploring differences by self-report versus objectively measured physical activity in high SES groups

Post-hoc exploration of active versus inactive control

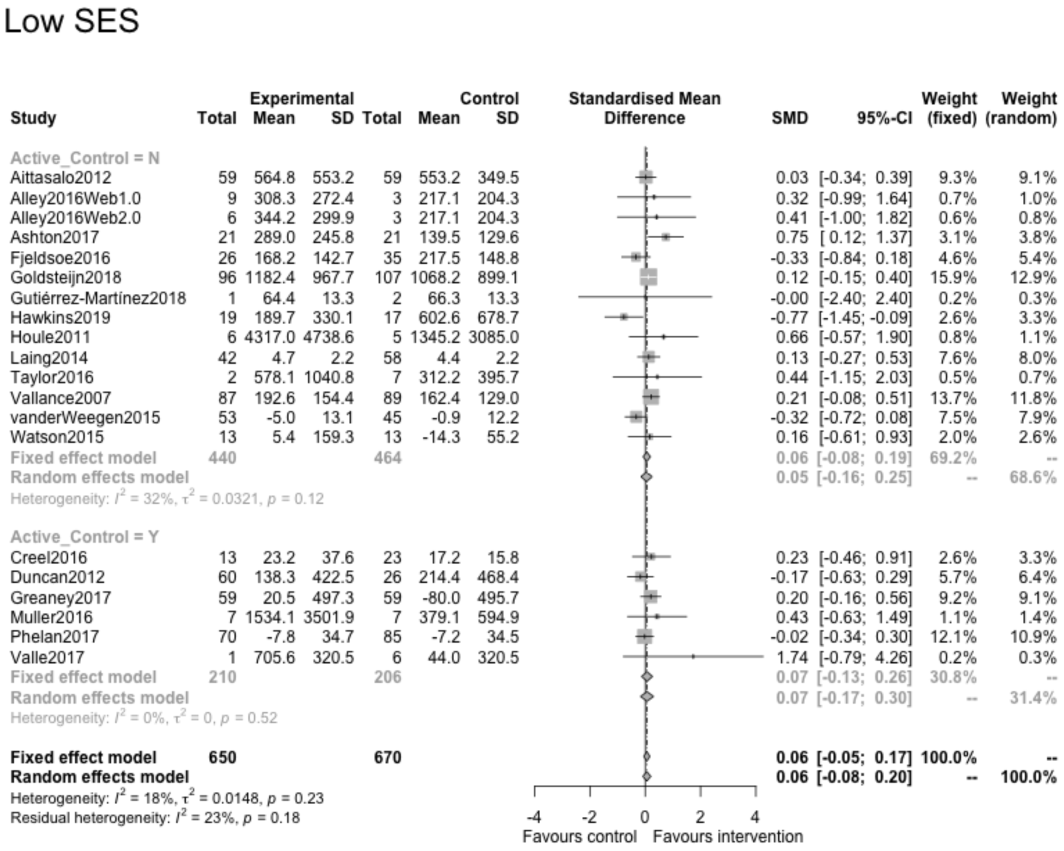

Figure 31: Post-hoc sensitivity analysis exploring differences by active versus inactive controls in low SES groups

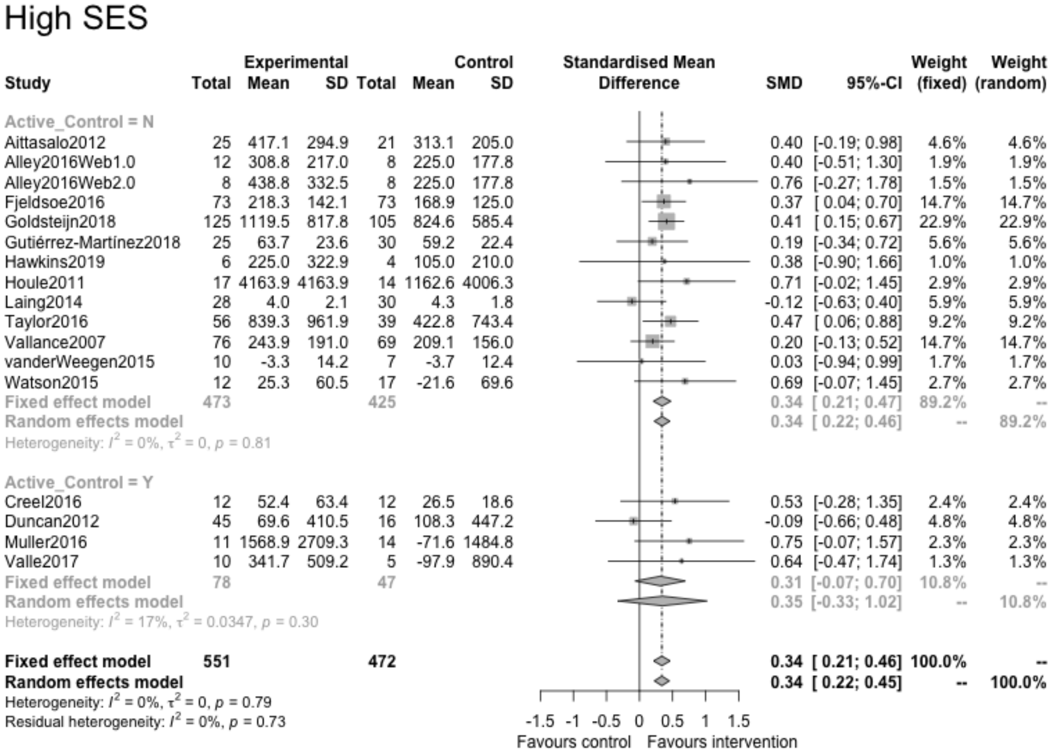

Figure 32: Post-hoc sensitivity analysis exploring differences by active versus inactive controls in high SES groups
